# Prioritization of schizophrenia risk genes from GWAS results by integrating multi-omics data

**DOI:** 10.1038/s41398-021-01294-x

**Published:** 2021-03-17

**Authors:** Dan He, Cong Fan, Mengling Qi, Yuedong Yang, David N. Cooper, Huiying Zhao

**Affiliations:** 1 Department of Medical Research Center, Sun Yat-Sen Memorial Hospital, Guangzhou, China; 2 Guangdong Provincial Key Laboratory of Malignant Tumor Epigenetics and Gene Regulation, Guangzhou, China; 3 School of Data and Computer Science, Sun Yat-Sen University, 510006 Guangzhou, China; 4 Institute of Medical Genetics, Cardiff University, Heath Park, Cardiff, CF14 4XN UK

**Keywords:** Genetics, Neuroscience

## Abstract

Schizophrenia (SCZ) is a polygenic disease with a heritability approaching 80%. Over 100 SCZ-related loci have so far been identified by genome-wide association studies (GWAS). However, the risk genes associated with these loci often remain unknown. We present a new risk gene predictor, rGAT-omics, that integrates multi-omics data under a Bayesian framework by combining the Hotelling and Box–Cox transformations. The Bayesian framework was constructed using gene ontology, tissue-specific protein–protein networks, and multi-omics data including differentially expressed genes in SCZ and controls, distance from genes to the index single-nucleotide polymorphisms (SNPs), and de novo mutations. The application of rGAT-omics to the 108 loci identified by a recent GWAS study of SCZ predicted 103 high-risk genes (HRGs) that explain a high proportion of SCZ heritability (Enrichment = 43.44 and $p = 9.30 \times 10^{ - 9}$). HRGs were shown to be significantly ($p_{\mathrm{adj}} = 5.35 \times 10^{ - 7}$) enriched in genes associated with neurological activities, and more likely to be expressed in brain tissues and SCZ-associated cell types than background genes. The predicted HRGs included 16 novel genes not present in any existing databases of SCZ-associated genes or previously predicted to be SCZ risk genes by any other method. More importantly, 13 of these 16 genes were not the nearest to the index SNP markers, and them would have been difficult to identify as risk genes by conventional approaches while ten out of the 16 genes are associated with neurological functions that make them prime candidates for pathological involvement in SCZ. Therefore, rGAT-omics has revealed novel insights into the molecular mechanisms underlying SCZ and could provide potential clues to future therapies.

## Introduction

Schizophrenia (SCZ) is a mental condition with a very complex etiology and highly variable clinical manifestations^1^. Although the disease has been studied for over a century, its underlying pathogenetic mechanisms remain unclear. Recently, two genome-wide association studies (GWAS) were performed on SCZ in an attempt to explore the etiology of the disease; together, they successfully identified over 100 SCZ-related loci^2,3^, although the identified GWAS loci mostly failed to identify any SCZ risk genes. It is however often difficult to interpret the functional links between the identified single-nucleotide polymorphisms (SNPs) and associated genes, especially when SNPs are located within noncoding regions. SNPs are generally considered to affect the expression of neighboring genes and therefore the genes in close proximity tend to be regarded as risk genes. Obviously, this ignores the fact that gene expression may be influenced by long-range regulators remote from their transcription start sites^4–6^.

To identify risk genes regulated by GWAS loci, many methods have been proposed. Most of these approaches have attempted to define candidate genes by setting a fixed distance around each index SNP and subsequently identifying SCZ risk genes by integrating genomic functions^7,8^, or considering topologically associated domains that are generated by prior chromatin interaction experiments^9,10^. A recent study has explored the gene regulatory mechanisms underlying SCZ by integrating functional genomics and position weight matrix (PWM)^7^ data. This study performed an in-depth analysis of the genome-wide protein binding landscape and PWM data to infer potential candidate genes for SCZ. Meanwhile, a web-based platform, FUMA, has been presented to provide gene-based functional annotation of GWAS results by accommodating positional, expression quantitative trait loci, and chromatin interaction mapping^11^. By integrating gene expression in the brain and chromosome conformation information^12^, Pardiñas et al. identified 42 potentially causal genes for SCZ. Another study employed a transcriptome-wide association study (TWAS) and successfully identified 157 TWAS-significant genes for SCZ, among which 42 genes were associated with specific chromatin features as measured in independent samples^13^. Taken together, these methods constitute a systematic framework to predict SCZ risk genes by integrating gene expression data with GWAS data.

Recently, the iRIGS method was proposed to identify risk genes in SCZ by integrating GWAS data with multi-omics data, including gene interaction and regulation networks, variant information, and differentially expressed genes^14^. In order to effectively integrate the data, a Bayesian network was employed in combination with Mahalanobis transformation^15^. However, the Mahalanobis transformation is applicable to the input matrix with a sample size larger than the number of features or non-singular covariance matrix. In addition, the iRIGS method was constructed using gene–gene networks based solely on gene ontology (GO) information without considering tissue-specific interactions between genes. Understanding the interactions between genes is a key step toward discovering new disease risk genes because the variants identified by genome sequencing are not independently associated with the disease, but they do interact with each other to form a systematic network as illustrated by many studies performed to date^16–18^. BioGRID is a commonly used database that lists interactions between proteins, including physical interactions and genetic interactions validated by 28 experimental systems^19^. Because of the importance of tissue specificity in protein–protein interaction (PPI), a database, TissueNet, was constructed by associating experimentally identified PPIs with human tissues^20^. Integrating the PPI information with other genomic features has the potential to significantly improve the prediction of disease risk genes.

**Fig. 1:**
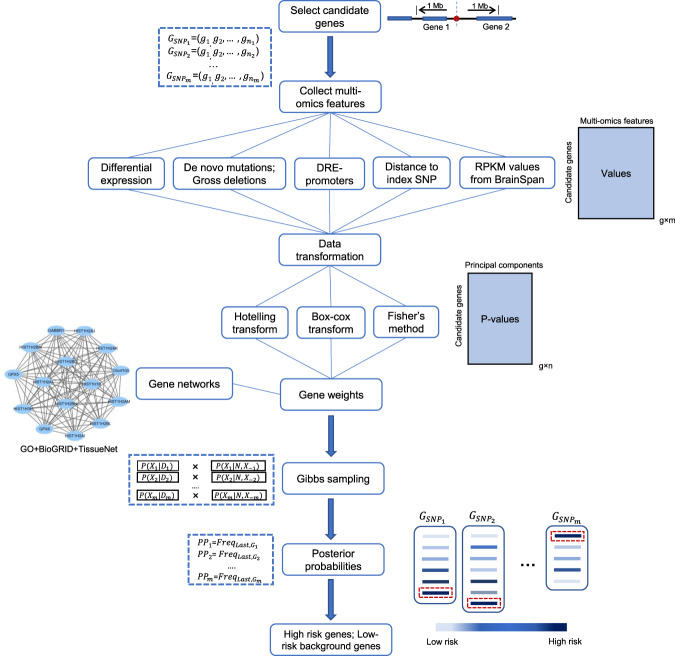
Schematic of the rGAT-omics framework. Candidate genes were initially defined as genes located within 1 Mb of SNPs associated with SCZ. The multi-omics data characterizing each gene were integrated by means of the Hotelling transform, the Box–Cox transformation, and Fisher’s method (Fisher’s combined probability test). The candidate genes were mapped to GO and PPI networks. The conditional probability of each candidate gene being a risk gene was then calculated based on the integrated multi-omics features and moving probability of selected genes in the GO and PPI networks obtained by a random walk with restart (RWR). One candidate gene with known conditional probability was sampled by Gibbs sampling to calculate the selecting frequency ${\mathrm{Freq}}_i$; the sampling was then continued until the frequency difference $\left\| {{\mathrm{Freq}}_{i + 1}-{\mathrm{Freq}}_i} \right\|$ was lower than $E_{{\mathrm{Gibbs}}} = 0.01$, which yielded the posterior probability (PP) for each candidate gene. For each locus, the candidate gene with the highest PP was considered to be the high-risk gene (HRG), whereas the candidate genes with PP values lower than the median value for all candidate genes were defined as low-risk background genes (LBGs). $D_l$ denotes the multi-omics features of gene *l*; *N* denotes GO and PPI networks.

Here, we developed a new method, rGAT-omics, to predict high-risk genes (HRGs) for a given disease. This method employs gene networks including the GO network, BioGRID network, and tissue-specific PPI network, and a combination of the Hotelling and Box–Cox transformations to integrate multi-omics data under a Bayesian framework (Fig. 1). Its application to SCZ identified 103 HRGs, which were shown to explain a significant proportion of SCZ heritability and were specifically expressed in brain tissues and SCZ-associated brain cell types. Among the genes, 16 HRGs had not been previously known to be associated with SCZ. Thus, the novel SCZ risk genes may provide new avenues for understanding the molecular basis of SCZ and exploring potential therapies.

## Results

### rGAT-omics identified HRGs by integrating multi-omics data with networks

rGAT-omics was developed by integrating multi-omics features (differential expression (DE), de novo mutations (DNM), gross deletions, distal regulatory elements (DRE) promoters, distance to index SNP (DTS), and Reads Per Kilobase of transcript per Million (RPKM) in adolescence and adulthood from BrainSpan) with the gene interaction networks including the GO network, BioGRID network^19^, and tissue-specific network from TissueNet^20^ (Fig. 1). Detailed information on the multi-omics features employed is given in Supplementary materials (Figs. S1 and S2). The application of rGAT-omics to 108 loci associated with SCZ provided by the previous GWAS study^2^ yielded 103 HRGs and 849 low-risk background genes (LBGs).

Among the 103 HRGs, 38 genes (36.9%) were the nearest to the index SNPs, while the remaining 65 HRGs were termed “non-nearest genes.” The non-nearest genes represent risk genes linked to 64 loci. For these loci, we collected the nearest genes to them to form the nearest gene set. The gene enrichment analysis was then performed on both the non-nearest and the corresponding nearest genes. As is evident from Table S1, the non-nearest genes were enriched in three gene sets (genes related to postsynaptic density(PSD), presynaptic proteins (PRP), and presynaptic active zone (PRAZ)) compared with the nearest genes. The information of the gene sets was shown in Table S2.

Compared to 849 LBGs, 103 HRGs were highly expressed in 13 brain tissues from the Genotype-Tissue Expression (GTEx) database and four brain regions from the BrainEAC database according to the tissue-specificity test (Fig. S3a, b). Specifically, the HRGs were highly expressed in the temporal cortex, frontal cortex, hippocampus, and occipital cortex brain regions. These brain regions have been shown in previous studies^21–24^ to be potentially associated with SCZ. A further test of the specificity of the HRGs in brain cell types found that the HRGs were specifically expressed in seven brain cell types as compared to the LBGs (Fig. S3c). Among them, four cell types, namely pyramidal cells (somatosensory cortex), pyramidal cells (hippocampus CA1), medium spiny neurons, and cortical interneurons, were associated with SCZ according to a recent single-cell study on cell types and GWAS signals of SCZ^25^. Additional enrichment analyses of gene sets showed that HRGs were significantly enriched in ten gene sets compared to the LBGs (Fig. S3d).

**Fig. 2:**
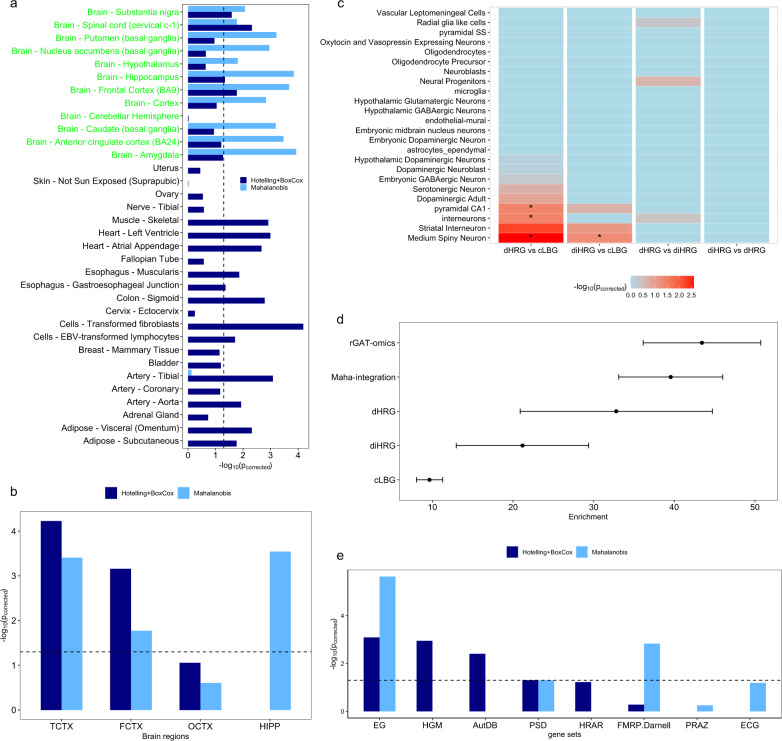
Comparing dHRGs and diHRGs in terms of tissue specificity, SCZ-related gene set enrichment, and cell specificity. **a** In GTEx data, diHRGs (*n* = 25) were highly expressed in 11 brain tissues, whereas dHRGs (*n* = 25) were highly expressed in four brain tissues compared to cLBGs (*n* = 641). Tissues names in green indicate brain tissues. **b** Compared to cLBGs, diHRGs were highly expressed in three brain regions, whereas dHRGs were highly expressed in two brain regions. **c** The cell-specificity analysis showed that dHRGs were specifically expressed in four cell types and three were associated with SCZ, whereas diHRGs were specifically expressed in one cell type and it was SCZ related compared to cLBGs. “*” denotes SCZ related cell type. dHRGs or diHRGs showed no significant variation in expression in any SCZ-related cell type compared to diHRGs or dHRGs. “dHRG vs cLBG” represents cell-specificity analysis on dHRGs using cLBGs as background genes; “diHRG vs cLBG” represents cell-specificity analysis on diHRGs using cLBGs as background genes; “dHRG vs diHRG” represents cell-specificity analysis on dHRGs using diHRGs as background genes; “diHRG vs dHRG” represents cell-specificity analysis on diHRGs using dHRGs as background genes. **d** The HRGs predicted by rGAT-omics represent a higher proportion of SCZ heritability compared to the HRGs predicted by Maha integration. Moreover, dHRGs explained higher proportion of SCZ heritability than diHRGs and cLBGs. The center values represent the enrichment and the error bars indicate standard errors. **e** dHRGs were significantly enriched in four gene sets, while diHRGs were enriched in three gene sets compared to cLBGs.

The proportion of SCZ heritability explained by HRGs was calculated by stratified linkage disequilibrium score regression^26^ (LDSC, https://github.com/bulik/ldsc/wiki/LD-Score-Estimation-Tutorial). We downloaded the SCZ summary statistics on 33,640 cases and 43,456 controls from the Psychiatric Genomics Consortium and plink files of 1000 Genomes Phase 3 from https://data.broadinstitute.org/alkesgroup/LDSCORE/. Since the distance to the index SNP represents a confounder in this regression, the risk genes used here were obtained without using DTS. We used SNPs within a 20 kb window center at the transcription start site of each HRG for LDSC analysis. We observed that SNPs close to the HRGs identified by rGAT-omics explained a large proportion of SCZ heritability with Enrichment = 43.44 and $p = 9.30 \times 10^{ - 9}$ (Fig. 2d). The enrichment was calculated by the equation: ${\mathrm{Enrichment}} = \frac{{h_{\mathrm{HRG}}^2/h_{All}^2}}{{{\mathrm{SNP}}_{\mathrm{HRG}}/{\mathrm{SNP}}_{\mathrm{All}}}}$, where $h_{\mathrm{HRG}}^2$ and $h_{\mathrm{All}}^2$ represent the heritability explained by SNPs around HRGs and by all SNPs in 1000 Genomes Phase 3, respectively, and where ${\mathrm{SNP}}_{\mathrm{HRG}}$ and ${\mathrm{SNP}}_{\mathrm{All}}$ denote the number of SNPs around HRGs and the total number of SNPs in 1000 Genomes Phase 3, respectively.

The involvement of 103 HRGs in biological functions was explored by the enrichment test of the biological processes functions. As a result, 45 functions were found to be significantly ($p_{\mathrm{adj}} < \, 0.05$) enriched by the HRGs, which included 12 functions involving neuronal or brain activities (Fig. S4 and Table S3). The detailed results are given in the Supplementary materials and Table S4. Moreover, we examined the involvement of the HRGs in terms of being targets for nervous system drugs (Supplementary materials, Table S5); 24 (23.3%) HRGs were identified as constituting targets for 4054 nervous system drugs. The HRGs are more enriched in drug targets for nervous system drugs than LBGs with odds ratio = 2.25 and $p = 1.90 \times 10^{ - 3}$. These results suggest the potential involvement of the HRGs in the etiology of SCZ.

### Effects of the Hotelling and Box–Cox transformations

rGAT-omics was constructed by integrating multi-omics features of genes through the Hotelling and Box–Cox transformations. These transforming approaches could be applied to non-singular covariant matrices that are not applicable to the Mahalanobis transformation as used in the previous study^14^. When the Mahalanobis transformation was used in rGAT-omics, the approach was termed Maha integration. The HRGs predicted by Maha integration were compared to the HRGs predicted by rGAT-omics. Maha integration predicted 103 HRGs. Among them, 25 were missed by rGAT-omics. Meanwhile, Maha integration missed 25 HRGs predicted by rGAT-omics. The HRGs missed by Maha integration were termed dHRGs, whereas the HRGs missed by rGAT-omics were termed diHRGs. Both rGAT-omics and Maha integration provided 641 common LBGs that were termed cLBGs.

Using cLBGs as background genes, we compared the tissue specificity of dHRGs and diHRGs. The tissue-specificity tests indicated that dHRGs were highly expressed in four brain tissues from GTEx, and two brain regions from BrainEAC. By contrast, diHRGs were specifically expressed in 11 brain tissues from GTEx and three brain regions from BrainEAC, as shown in Fig. 2a, b. Thus, the HRGs predicted by Maha integration are more likely to be highly expressed in brain tissues.

The specificity analysis on brain cell types indicated that dHRGs were specifically expressed in four brain cell types including three cell types previously shown to be SCZ associated^25^. In comparison, diHRGs were specifically expressed in only one cell type, which has been previously indicated to be SCZ associated (Fig. 2c). Thus, the HRGs predicted by rGAT-omics are more likely to be highly expressed in SCZ-associated brain cell types.

We observed that SNPs close to the HRGs identified by rGAT-omics explained a proportion of SCZ heritability with Enrichment = 43.44 and $p = 9.30 \times 10^{ - 9}$. In comparison, SNPs close to the HRGs identified by Maha integration explain a significant enrichment of SCZ heritability with Enrichment = 39.56 and $p = 7.04 \times 10^{ - 9}$. Using the same strategy, we also compared the heritability explained by SNPs close to dHRGs and diHRGs. The result indicated that the SNPs close to dHRGs (Enrichment = 32.81, $p = 7.40 \times 10^{ - 3}$) explained a higher enrichment of SCZ heritability than the SNPs close to diHRGs (Enrichment = 21.18, $p = 0.015$). More detailed results are shown in Fig. 2d. Thus, the HRGs predicted by integrating multi-omics data and combining Hotelling and Box–Cox transformation represent a higher proportion of SCZ heritability than using the Mahalanobis transformation.

Another comparison to be made was the enrichment of HRGs in genes expressed significantly differently between SCZ patients and controls. Among 25 dHRGs, eight (32.0%) were found to be expressed significantly different between patients and controls. By contrast, only two (8.0%) diHRGs were found to be expressed significantly differently between SCZ patients and controls, which is much lower than dHRGs predicted by rGAT-omics (*p* = 0.037). Thus, the HRGs predicted by a combination of Hotelling and Box–Cox transformation are more likely to be expressed significantly differently between SCZ patients and controls. In terms of gene set enrichment, we found that dHRGs are enriched in EG (Essential Genes), HGM (Human Gene Module), AutDB, and PSD (PostSynaptic Density) gene sets as compared to cLBGs, whereas diHRGs are enriched in three gene sets, EG, PSD, and FMRP.Darnell (Fig. 2e).

When simply substituting the Mahalanobis transformation with the combination of Hotelling and Box–Cox transformations in the iRIGS model, we compared the predicted HRGs by two transformation approaches. The approach using a combination of Hotelling and Box–Cox transformations is termed HB-transformations. Using this HB transformation, 105 genes were predicted as HRGs. In comparison, the Mahalanobis transformation predicted 104 HRGs. Of the 105 HRGs, 17 were not identified by the Mahalanobis transformation. Of the 104 HRGs predicted by the Mahalanobis transformation, 16 were missed by HB transformation. The HRGs missed by the Mahalanobis transformation were found to be enriched in two gene sets, PSD^27^ and EG^28^ with one-sided Fisher’s exact test *p* values, $4.90 \times 10^{ - 3}$ and 0.017, respectively, compared to 704 common LBGs identified by two methods. PSD is a gene set including genes involving in synaptic and presynaptic functions, and EG is a gene set collecting autism-relevant genes. The detailed information on the gene sets is given in the “Methods” section and Table S2. However, the HRGs predicted by the Mahalanobis transformation but missed by HB transformation were only enriched in the gene set EG ($p = 1.53 \times 10^{ - 3}$) compared to common LBGs. Table S6 shows the results of the gene set enrichment test on 15 different gene sets.

Further examination revealed the enrichment of HRGs expressed significantly different in SCZ patients and controls. We found that seven (41.2%) of the HRGs missed by the Mahalanobis transformation were differentially expressed in SCZ patients compared to controls. By contrast, only two (12.5%) of the HRGs missed by HB transformation were differentially expressed in SCZ patients and controls. Using the Hotelling and Box–Cox transformations together improves the prediction over that using the Mahalanobis transformation alone.

In summary, the HRGs predicted by combining Hotelling and Box–Cox transformations explained more SCZ heritability, are more likely to be expressed in SCZ-associated cell types, and are more likely to be expressed significantly differently between SCZ patients and controls than the HRGs predicted by the method using only the Mahalanobis transformation.

### The necessity of combining networks in the prediction

**Fig. 3:**
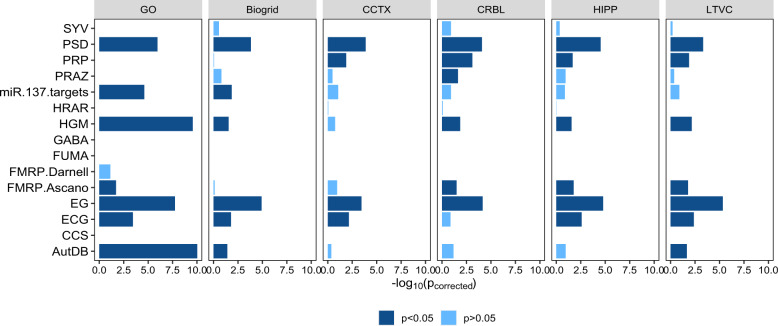
Gene set enrichment analysis of HRGs predicted by rGAT-net. The enrichment analysis was performed by one-sided Fisher’s exact test with Bonferroni correction. The dark blue bar represents the enrichment test *p* value < 0.05 in one gene set and the light blue bar represents the enrichment test *p* value > 0.05 in one gene set dataset. The full name and brief description of each dataset are given in Table S2. HRGs predicted by the GO and BioGRID networks are enriched in miR-137 targets that are not enriched by using any networks from the TissueNet database in the prediction. HRGs predicted by TissueNet are all enriched in PRP that are not enriched by the HRGs predicted by using networks from GO and BioGRID. Only HRGs predicted by the CRBL network are enriched in gene set PRAZ.

As shown in Table 1 and Fig. 1, rGAT-omics integrated GO, BioGRID, and TissueNet networks with multi-omics data to allow the inference of HRGs. To assess the contributions of the networks in the prediction, we performed rGAT-omics by networks without integrating other features. This modified approach using only the networks was termed rGAT-net. rGAT-net was run in six versions, namely GO network, BioGRID network, and four types of tissue-specific PPI network including the cerebral cortex, cerebellum, hippocampus, and lateral ventricle, respectively. The numbers of HRGs and LBGs identified in each version are shown in Table S7. The six versions of rGAT-net were directly compared in terms of the enrichment of HRGs in the gene sets. As shown in Fig. 3, the HRGs predicted by the networks from TissueNet were all significantly enriched in genes related to PRP^29^ that were not enriched by the HRGs predicted by the GO network or the BioGRID network. In addition, the PRAZ^29^ dataset was only enriched by the HRGs predicted by the cerebellum network from TissueNet. By contrast, the HRGs predicted by the GO and BioGRID networks were enriched in miR-137 targets^30^ that were not enriched by any networks from TissueNet. Thus, it was necessary to integrate the GO network, BioGRID network, and networks from TissueNet in the predictive algorithm.

**Table 1:** Transformation approaches, networks, and multi-omics features used by rGAT-omics.

	rGAT-omics
Transformation	Hotelling transformation and Cox–Box transformation
Networks	GO and PPI networks from BioGRID and brain tissue
Multi-omics features	DE^a^, DNM^b^, DRE promoters^c^, DTS^d^, gross deletions, and RPKM^e^ in adolescence and adulthood from BrainSpan

^a^*P* values of differential gene expression analysis in SCZ patients and controls.

^b^Probability of genes with de novo mutations being carried by SCZ patients.

^c^Distal regulatory elements obtained from CapHiC, FANTOM5, BrainCP, and BrainGZ databases.

^d^Distance to index SNP.

^e^Reads Per Kilobase of transcript per Million mapped reads from BrainSpan.

**Fig. 4:**
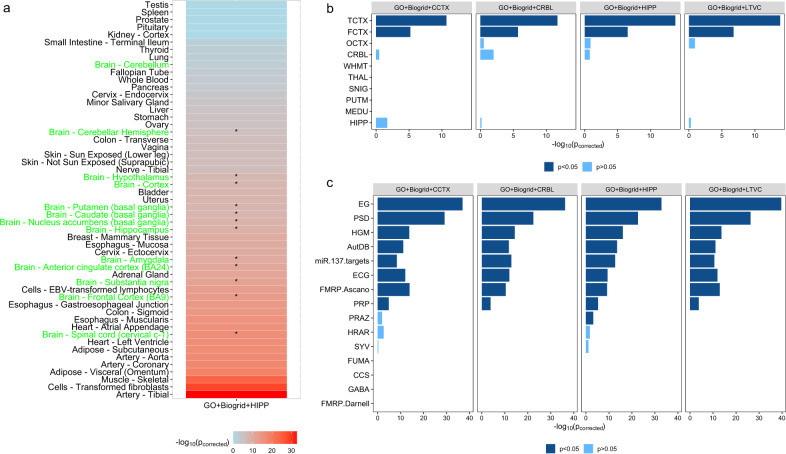
Comparing the tissue specificity of HRGs and LBGs predicted by integrating GO, BioGRID, and four types of tissue-specific PPI network. **a** HRGs predicted by four forms of network integration are all highly expressed in the same brain tissues compared to LBGs. “*” denotes that the predicted HRGs are significantly enriched in the brain tissues. Tissue names in green represent brain tissues. **b** HRGs predicted by four forms of network are all highly expressed in the same two brain regions compared to LBGs. **c** HRGs predicted by GO and PPI networks from BioGRID and hippocampus are enriched in nine SCZ-related gene sets, and HRGs predicted by other forms of network are enriched in eight SCZ-related gene sets.

We then integrated the GO network, the BioGRID network, and the networks from TissueNet. The number of predicted HRGs and LBGs is shown in Table S7. The HRGs predicted by integration of the networks are all highly expressed in the 12 brain tissues from the GTEx dataset (Fig. 4a) according to the tissue-specificity analysis described in the “Methods.” However, the HRGs predicted only by means of the GO network show no significant enrichment in any brain tissue from the GTEx dataset (Table S8). Tissue-specificity analysis further indicated that the HRGs predicted by the integration of networks were highly expressed in two brain regions obtained from the BrainEAC database (Fig. 4b). By contrast, the HRGs identified by the GO network were highly expressed in one brain region obtained from the BrainEAC database (Table S8). As shown in Fig. 4c, the HRGs identified by integrating GO with PPIs from the BioGRID and hippocampus networks were enriched in nine gene sets, whereas the HRGs predicted by other forms of integration were enriched in eight gene sets.

Although PPI networks from different brain tissues make similar contributions, integrating the network from the hippocampus with the GO network and the BioGRID network gave the best performance (Fig. 4). This network predicted 106 HRGs and 828 LBGs. The HRGs predicted by this network were examined in relation to their multi-omics features, including differently expressed (DE) genes, DNMs, gross deletions, and DRE promoters. The detailed results are shown in the Supplementary materials. We found that the multi-omics features of the HRGs and LBGs predicted by the integrated networks consistently exhibited evidence to support the higher risks of the HRGs in SCZ than the LBGs.

### Comparing rGAT-omics with other approaches

We compared the HRGs predicted by rGAT-omics with the risk genes obtained by other approaches. A recent TWAS on SCZ^13^ has been developed to implicate SCZ-associated genes. In total, this study identified 157 unique TWAS-significant genes for SCZ, in which ten were predicted as HRGs by rGAT-omics. The overlap represented a significant enrichment compared to chance alone (binomial test $p = 1.1 \times 10^{ - 8}$). Moreover, the number of overlapping genes is significantly greater than for the LBGs (one-sided Fisher’s exact test $p = 0.031$, ${\mathrm{OR}} = 2.23$).

Another GWAS study of SCZ presented 42 genes as being potentially casual genes for SCZ^12^. Among these genes, four were predicted to be HRGs by rGAT-omics, which represents a significant enrichment compared to chance alone (binomial test $p = 6.7 \times 10^{ - 5}$). Compared to LBGs, the number of overlapping genes is significantly elevated (one-sided Fisher’s exact test $p = 6.43 \times 10^{ - 3}$, ${\mathrm{OR}} = 8.50$).

Finally, we compared rGAT-omics with iRIGS. iRIGS predicted 104 genes as HRGs. Among them, 49 overlapped with the HRGs predicted by rGAT-omics. This overlap is significant compared to chance alone with a binomial test $p \, < \, 2.2 \times 10^{ - 16}$. By contrast, two LBGs predicted by rGAT-omics were identified as HRGs by iRIGS, which is significantly lower than the number that overlapped with the HRGs (one-sided Fisher’s exact test, $p = 5.51 \, \times 10^{ - 16}$, ${\mathrm{OR}} = 44.71$).

### Novel HRGs predicted by rGAT-omics

**Fig. 5:**
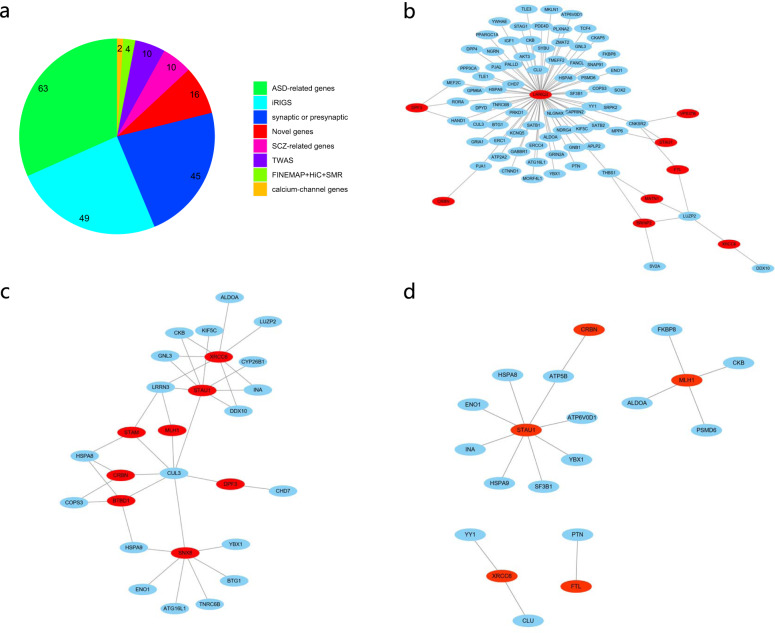
The HRGs predicted by rGAT-omics exhibited partial overlap with those in other datasets or predicted by other methods. **a** “ASD-related gene sets” represents genes associated with ASD and is from AutDB, ECG, EG, HGM, and HRAR sets (Table S2). In total, 5,138 genes were present in this set, while 63 were predicted to be HRGs by rGAT-omics. “iRIGS” represents HRGs predicted by the iRIGS method. In all, 104 HRGs were predicted by iRIGS, which included 49 already predicted by rGAT-omics. “Synaptic or presynaptic genes” denotes a set of genes involving synaptic or presynaptic functions, which are from FMRP, PRAZ, PRP, PSD, and SYV datasets as shown in Table S2. In total, 2,803 genes were included in this dataset, which include 45 predicted as the HRGs by rGAT-omics. “SCZ-related genes” represent genes from GABA or miR.137.targets gene sets or FUMA (Table S2). In total, 145 genes are included in this dataset, in which ten were predicted as HRGs by rGAT-omics. “TWAS” represents a TWAS study for SCZ that identified 157 SCZ-associated genes, of which ten overlaps with the HRGs predicted by rGAT-omics. “FINEMAP+HiC+SMR” represents SCZ causal genes identified by integrating FINEMAP, chromosome conformation, and SMR analysis. In total, 42 genes were predicted to be SCZ associated and four of them were predicted to be HRGs by rGAT-omics. “Calcium channel genes” denotes genes from CCS dataset that include 73 genes. Among them, two were predicted as HRGs by rGAT-omics. “Novel genes” represent the HRGs predicted by rGAT-omics but not included in any other datasets or methods. **b** Red spots denote novel genes and blue spots denote non-novel genes. Nine novel genes interact with the non-novel genes in the GO database. Genes with >30% annotations in common were lined up. **c** Eight novel genes have >30% interactions shared with the non-novel genes in TissueNet (hippocampus). Genes with >30% interactions in common were lined up. **d** Five novel genes have 16 interactions with the non-novel genes in the BioGRID database.

Figure. 5a shows that the HRGs predicted by rGAT-omics overlapped with those genes present in other datasets or predicted by other algorithms. Here, 63 HRGs overlapped with ASD-related genes that were included in AutDB, ECG, EG, HGM, and HRAR (highest-ranking autism risk) datasets (Table S2). Moreover, 45 HRGs were included in the dataset containing synaptic or presynaptic genes from the PRAZ, PRP, PSD, and SYV datasets. In addition, ten genes overlapped with the SCZ-related gene set. This gene set includes 145 genes that are predicted to be SCZ related by FUMA, or collected in the GABA dataset or the miR.137.targets dataset (Table S2).

Importantly, 16 HRGs (Table 2 and Fig. 5a) were novel identifications in this study. As shown in Table 2, 13 of these 16 HRGs were not the nearest genes to the index SNP markers, illustrating precisely why these genes would have been difficult to identify as risk genes by conventional approaches. Ten out of the 16 genes have been reported as being associated with neurological disorders. The detailed functions of these genes are shown in the Supplementary materials.

**Table 2:** Novel SCZ risk genes predicted by rGAT-omics.

Gene symbol	Index SNP	Distance to index SNP (bp)
^▲^SNX8*	chr7_2025096_I	368,857
^▲^NDUFAF4*	rs117074560	886,106
^▲^*LRRIQ3*	rs12129573	895,505
^▲^*MATN1*	rs1498232	762,483
^▲^CRBN*	rs17194490	673,608
DPF3*	rs2332700	943,483
^▲^VPS37B*	rs2851447	284,122
^▲^FTL*	rs56873913	622,633
XRCC6*	rs9607782	429,567
RPRD1B*	rs6065094	791,246
^▲^*BRINP2*	rs6670165	140,058
^▲^STAU1*	rs7267348	326,132
^▲^NCAPD3*	rs75059851	272,779
MLH1*	rs75968099	176,240
STAM*	rs7893279	1,058,981
BTBD1*	rs950169	970,355

*The gene is not the nearest gene to the index SNP among all candidate genes.

^▲^The gene is reported as being associated with neurological functions.

We also explored the interactions between the 16 novel genes and 87 other genes, termed non-novel genes in the GO network, gene network from TissueNet, and protein network from BioGRID. As shown in Fig. 5b, nine of the novel genes connect with 74 non-novel genes through GO annotation. This network includes 87 interactions, which is significant compared to random chance (permutation test $p = 2.8 \times 10^{ - 3}$). Here, a novel gene was defined as interacting with a non-novel gene if the shared GO annotations between them was >30%. The permutation test was performed by randomly sampling 16 genes from the complement of human genes and counting the number of interactions between them and the non-novel genes. This process was repeated 10,000 times to calculate an experience *p* value based on the number of interactions. Additional analysis was performed to evaluate the interactions between the novel genes and the non-novel genes in TissueNet network. As shown in Fig. 5c, eight novel genes interacted with 19 non-novel genes in TissueNet, which is significantly higher than random (permutation test, $p \, < 10^{ - 4}$). In the BioGRID network, 16 interactions were found between five of the novel genes and 15 non-novel genes (Fig. 5d), which is significantly higher than expected by chance alone (permutation test, $p \, < 10^{ - 4}$). These results are supportive of a relationship between the novel genes and SCZ. The novel genes involved in the networks are shown in Table S9.

## Discussion

SCZ is a chronic and severe complex mental disorder that affects >20 million people worldwide^31^. Because the underlying pathogenic mechanisms are not yet clear, SCZ can be suppressed and treated, but in most cases cannot be completely cured. Recently, GWAS studies have revealed many SNPs associated with SCZ. However, the impact of these SNPs on gene function is largely unknown. There are increasing evidences to support the contention that the risk genes are not necessarily those residing closest to the index SNP^4,32^. Clearly, identifying the risk genes is a prerequisite for revealing the molecular mechanisms underlying SCZ.

The dramatic increase in the availability of multi-omics data has provided a large body of information that is potentially very useful for linking SNPs to HRGs. For example, previous studies have located index SNPs to regulatory elements of known neuropsychiatric disorder genes through enhancer looping^4^. Other studies have highlighted the links between SNP markers and gene deletions^33,34^. Adding features such as transcriptomics, functional genomics, epigenomics, or more accurate genomic information promises to improve the accuracy of risk gene prediction. In this study, we developed a model, rGAT-omics, whose novelty relies on its integration of gene–gene and protein–protein networks with multi-omics data from different sources to predict risk genes using an unsupervised learning method. The networks used in this study were the GO network, PPIs from BioGRID, and PPIs from TissueNet. Integrating gene interactions from three very different sources provided an improvement in terms of the results over those obtained by using only one kind of network. Another novel aspect of rGAT-omics is that it used Hotelling and Box–Cox transformations to accommodate multifeatures with singular covariance matrix and integrating multi-omics features. When there are too many features or a large linear correlation between features, variable dimensionality reduction helps to remove redundant information and improve prediction accuracy. Comparison of two transformation approaches indicated that combining the Hotelling and Box–Cox transformations can identify risk genes (and help to explain a larger proportion of heritability) that are more likely to be expressed in SCZ-associated brain cell types than using Mahalanobis transformation alone.

The performance of the prediction was further evaluated by false-positive rate (FPR) calculated using simulation data. In this study, a total of 6,688 genes were used for functional enrichment analysis of the predicted genes. After excluding these genes from human genes, the simulated datasets were generated by sampling 6,688 genes from the remaining human genes for 1,000,000 times. The genes obtained from each simulation were used to evaluate the FPRs of two methods, rGAT-omics and iRIGS. These two methods computed posterior probability (PP) as association scores for all candidate genes with each SNP. The FPRs of both methods were calculated for genes with association scores ranked from top 1 to top 6. FPR was the number of predicted genes overlapped with the negative dataset divided by the total number of predicted genes. The significance of FPR difference for the two methods was evaluated by one-sided Wilcoxon’s rank-sum test. The result indicated that the FPRs of rGAT-omics were significantly lower ($p \, < 2.2 \times 10^{ - 16}$) than the FPRs of iRIGS (Fig. S5a).

In order to compare the per SNP heritability of HRGs predicted by rGAT-omics to LBGs, we calculated per SNP heritability (the proportion of phenotypic variation explained by the SNP) by BLD-LDAK Model within LDAK^35^. The average heritability of SNPs within a 20 kb window of the transcription start site of the predicted HRGs was compared to that of SNPs within 20 kb of the predicted LBGs in Fig. S5b. As shown in Fig. S5b, the average heritability of SNPs around HRGs is significantly ($p \, < 2.2 \times 10^{ - 16}$) higher than the average heritability of SNPs around LBGs.

One of the advantages of rGAT-omics is that it can detect genes not the nearest to the index SNP markers, which would have been difficult to identify as risk genes by conventional approaches. This methodology can in principle also be applied to other psychiatric disorders, and indeed any multifactorial condition with a genetic component, to predict risk genes by selecting corresponding genetic characteristics according to the question being posed. If we combine the links between psychiatric disorders, thereby giving more weight to the risk genes known to be related to other related diseases, the results could in principle be improved still further.

This method attributes each gene a PP associated with SCZ. The PP of genes can be used to select more than one risk gene for each locus by setting a threshold according to the number of candidate genes associated with each locus. However, the precise number of risk genes at each locus is unknown; hence, the selection of risk genes at each locus remains a challenge. Here, we provided candidate genes with PP ranked in the top 10% in Table S10.

Another shortcoming of this method is in defining candidate genes as genes within 1 Mb of index SNPs, an approach that ignores the actual three-dimensional distances between SNPs and genes. If genes within a certain distance of the three-dimensional space described by the locus can be chosen, the selection of candidate genes will become biologically and clinically more appropriate.

## Methods

### Constructing Bayesian model for prediction of risk genes

The goal of rGAT-omics was to probabilistically rank candidate genes at each GWAS locus based on their multi-omics supporting evidence and closeness in gene–gene networks. The framework of rGAT-omics is shown in Fig. 1. We selected genes in the $\pm$1 Mb region centered at a GWAS index SNP as candidates for that locus. Finally, we selected genes with the highest-ranking score in each locus. Let $L$ be the number of GWAS loci and $(X_1,X_2, \ldots ,X_L)$ be a set of candidate risk genes, each being selected from one of the *L* loci. We used $D$ to denote the genomics data for all candidate genes for all GWAS loci and $N$ to denote gene–gene networks. Now the goal was to calculate $P\left( {X_1, \ldots ,X_L{\mathrm{|}}D,N} \right)$ and selected $L$ risk genes, which maximize $P\left( {X_1, \ldots ,X_L{\mathrm{|}}D,N} \right)$. However, enumerating all possible gene combinations was infeasible. Thus, we used Gibbs sampling to transition the problem into a conditional single-dimensional sampling procedure.

According to Bayesian theory, $P\left( {X_1, \ldots ,X_L{\mathrm{|}}D,N} \right)$ can be calculate as Eq. (1).

1 $$\begin{array}{*{20}{c}} {P\left( {X_1, \ldots ,X_L{\mathrm{|}}D,N} \right) = \mathop {\prod }\limits_{l = 1}^L P\left( {X_l{\mathrm{|}}X_{ - l},N} \right)P\left( {D_l{\mathrm{|}}X_l} \right)} \end{array}$$

As shown in Eq. (1), the association probability of each candidate risk gene from locus $l$ was composed of two terms, $P\left( {D_l{\mathrm{|}}X_l} \right)$ and $P\left( {X_l{\mathrm{|}}X_{ - l},N} \right)$, that represent the genomics features of gene $l$, and the complex correlations of genes with other candidate risk genes, respectively. $X_{ - l}$ denotes a vector of candidate risk genes with the $l$th gene removed.

Here, $P\left( {X_l{\mathrm{|}}X_{ - l},N} \right)$ represents a Bayesian factor on correlations of a gene with other candidate risk genes $X_{ - l}$ through network $N$. A gene from locus $l$ closer (larger edge weight) to $X_{ - l}$ was more likely to be a risk gene compared to other candites from the same locus. The distance between $X_l$ and $X_{ - l}$ is calculated by a random walk with restart algorithm (Supplementary materials).

The other Bayesian factor, $P\left( {D_l{\mathrm{|}}X_l} \right)$, was calculated by employing Hotelling transform and Box–Cox transform. Each gene was represented by collected multi-omics features that could be separated into two categories. The first category is features represented by *p* values including DE, DNMs, and gross deletions, and the second are features not represented by *p* values including DTS, DRE promoters, and RPKM in adolescence and adulthood from BrainSpan. In order to combine these two categories, we used the Hotelling and Box–Cox transformations to convert the second feature categories into *p* values for a combination. For a given feature matrix $P \in {\Bbb R}^{g \times m}$ with $g$ genes and $m$ features, the Hotelling transform is performed as

2 $$\begin{array}{*{20}{c}} {P^{\prime} = U\left( {P - M} \right)^{\mathrm{T}}} \end{array}$$

where $V = \left[ {v_1;v_2; \ldots ;v_m} \right]$ are eigenvectors for the covariance matrix of $P$ corresponding to decreasing eigenvalues with $\lambda _1 \ge \lambda _2 \ge \cdots \ge \lambda _m$. $M$ is the column mean of *P*, and $U = \left[ {v_1;v_2; \ldots ;v_n} \right]^{\mathrm{T}}$with $n \le m$ as the number of chosen principal components.

The Box–Cox transformation is

3 $$\begin{array}{*{20}{c}} {pi^{\prime} = \left\{ {\begin{array}{*{20}{c}} {\frac{{pi^{\beta _i} - 1}}{{\beta _i}},\;\beta _i \, \ne \, 0} \\ {{\mathrm{log}}\left( {pi} \right),\;\beta _i = 0} \end{array}} \right.} \end{array}$$

where $pi$ is the $i$th row vector of $P^{\prime}$ and should be made all elements positive by adding a constant; $\beta _i$ is the optimal transformation parameter for $pi$.

Then, we standardized each $pi{\prime}$ and calculated their *p* values in a Gaussian distribution. For each gene $X_l$, we combined its *p* values from two categories by Fisher’s method (Fisher’s combined probability test) as

4 $$\begin{array}{*{20}{c}} {P\left( {D_l{\mathrm{|}}X_l} \right) = - \ln \left( {\chi _{n + n^{\prime}}^2{\,}^{ - 1}\left( { - 2\left( {\mathop {\sum}\limits_{i = 1}^n {\log \left( {pi_l^\prime } \right)} + \mathop {\sum}\limits_{j = 1}^{n{\prime}} {\log \left( {p_{l_j}} \right)} } \right)} \right)} \right)} \end{array}$$

where, $n$ is the number of *p* values from the second feature category attached to each gene and $pi_l^\prime$ is the $l$th element of $pi^\prime$, which is a vector obtained from Eq. (3); $n^{\prime}$ is the number of *p* values from the first feature category attached to each gene; $p_{l_j}$ is the $j$th *p* value of gene $l$ in the first feature category.

Finally, we applied Gibbs sampling to sample candidate risk genes for a given locus *l* to maximum $P_l = P(X_l|X_{ - l},N)P(D_l|X_l)$. We iterated the sampling process until the selected frequency (PP) of genes converged. Specifically, Gibbs sampling were performed in two cycles of iteration. In the first cycle of iteration, Gibbs sampling was initiated by selecting the genes for each locus with equal sampling probabilities. Then, the candidate risk gene for locus $l$ was sampled according to one-dimensional PP $P_l$, and then iterated across each locus. In the second cycle of iteration, Gibbs sampling was initiated with the candidate risk genes obtained from the last iteration of the first cycle. In both cycles, the selected frequency ($\frac{\# \,{\mathrm{of}}\,{\mathrm{times}}\,{\mathrm{the}}\,{\mathrm{gene}}\,{\mathrm{is}}\,{\mathrm{selected}}}{\# \,{\mathrm{of}}\,{\mathrm{sampling}}}$) of each gene was updated after each time of sampling. All selected frequencies of candidate genes in $i$th iteration were denoted as a vector, ${\mathrm{Freq}}_i$. When the Euclidean norm of ${\mathrm{Freq}}_i - {\mathrm{Freq}}_{i - 1}$ was smaller than $E_{{\mathrm{Gibbs}}}$ ($E_{{\mathrm{Gibbs}}}$ was set as 0.01), the iteration was stopped.

### Gene sets enrichment analysis

We downloaded 12 gene sets that were obtained on the basis that they were related to SCZ or other neurological disorders including autism. These gene sets (FMRP.Ascano, FMRP.Darnell, GABA, PRP, PRAZ, SYV, ECG, EG, miR.137.targets, PSD, AutDB, and CCS) were obtained from different sources as described in a previous study^14^, and in Table S2. In addition, we collected HRAR, genes associated with autism in HGM from SFARI^36^, and 84 risk genes for SCZ predicted by FUMA^11^. In total, 6,688 genes were collected in the gene sets. The number of genes included in each gene set is shown in Table S2. The proportions of genes that were overlapping between datasets are shown in Fig. S6. The gene set enrichment analysis was assessed by means of one-sided Fisher’s exact test with Bonferroni correction. All tests in this article were defined as being significant if the *p* values were < 0.05.

### HRGs and LBGs

We performed rGAT-omics on 108 loci reported in a previous GWAS^2^. All genes located within a 2 Mb window centered at the index SNP were defined as candidate genes. From these, the genes with PP values less than the median PP values of all candidate genes were defined as LBGs. A gene was defined as an HRG if its PP value was higher than that of any other candidate gene from the same locus. We predicted HRGs and LBGs after merging the overlapping genes across loci.

## Supplementary information

Supplementary materials
