# Supplementary material for: Prioritization of schizophrenia risk genes from GWAS results by integrating multi-omics data
Source: Transl Psychiatry. 2021 Mar 17;11:175. doi: 10.1038/s41398-021-01294-x (PMC7969765; doi:10.1038/s41398-021-01294-x)
Supplement: Supplementary file 1 — Supplementary materials [file 41398_2021_1294_MOESM1_ESM.pdf]

Supplementary materials

Methods

Experimental systems included in BioGRID database

BioGRID collates 28 experimental systems to provide evidence to support interactions between proteins. They are Two-hybrid, Affinity Capture-MS, Affinity Capture-Western, Dosage Rescue, Reconstituted Complex, Synthetic Growth Defect, Synthetic Lethality, Synthetic Rescue, Affinity Capture-Luminescence, Biochemical Activity, Co-crystal Structure, Far Western, FRET, Protein-peptide, Co-localization, Affinity Capture-RNA, Protein-RNA, PCA, Co-purification, Co-fractionation, Dosage Lethality, Phenotypic Enhancement, Phenotypic Suppression, Dosage Growth Defect, Negative Genetic, Positive Genetic, Synthetic Haploinsufficiency and Proximity Label-MS.

Multi-omics data

This study integrated multi-omics data to predict risk genes; these include distance to index SNP (DTS), differential expression (DE) of genes in SCZ patients and controls, distal regulatory element (DRE)-promoter links, *de novo* mutations (DNMs), gross deletions and developmental stage specificity expression of genes in brain regions.

DTS is the distance in base-pairs from the transcription start site of each gene to the position of the index SNP. Differential expression (DE) of gene values were obtained by analyzing RNA-seq data from the CommonMind Consortium comprising 258 subjects with SCZ and 279 controls[1]. Distal regulatory element (DRE)-promoter links were obtained from three sources: (i) 221,069 links for the cortical and subcortical plate and 228,323 links for the germinal zone[2]; (ii) another study[3] including 1.6 million promoter interactions that were generated by Hi-C on GM12878 cells; (iii) FANTOM5 cap analysis of gene expression (CAGE) atlas including 66,942 enhancer-promoter

links[4]. RNA sequencing data on the developmental stage specificity of expression of genes in brain tissues were downloaded from BrainSpan (https://www.brainspan.org). The gene expression level was calculated by the average expression of five brain regions (frontal lobe, temporal lobe, parietal lobe, occipital lobe and cerebellum) in two developmental stages (adolescence and adulthood). DNMs were downloaded from four previous studies[5-8], from which we extracted 698 DNMs with intragenic mutations including nonsense, splicing, frameshift and missense associated with SCZ. SCZ-related gross deletions were downloaded from the Human Gene Mutation Database (HGMD)[9]; in total, we obtained 149 gross deletions involving 73 genes.

All missing data in these data sources were filled in by means of the K-Nearest Neighbors algorithm.

Gene expression analysis on tissue and cell specificities

The tissue specificity levels of genes were calculated by a previously described strategy[10] and evaluated by a one-sided Wilcoxon rank-sum test with Bonferroni correction. Briefly, let $E = (E_{1,}E_{2,...,}E_T)$ be the expression vector of a given gene across all tissues, where T is the total number of tissues. Then, we normalized E by $E/sum(E)$. Let $E^t = (E_1^t, E_2^t, \dots E_i^t, \dots, E_T^t)$ be the expression of a gene only expressing in tissue $t, t = 1,2, \dots, T$. That is, $E_i^t = \begin{cases} 0, & i \neq t \\ 1, & i = t \end{cases}$. Next, we computed the entropy and Jensen-Shannon (JS) divergence of E and E^t. The entropy of a discrete distribution $p = (p_1, p_2, \dots, p_n)$ is calculated by $H(p) = -\sum_{i=1}^{n} p_i \ln{(p_i)}$, where $\sum_{i=1}^{n} p_i = 1$ and $0 \le p_i \le 1$. The JS divergence of two discrete distributions, p^1 and p^2, is $JS(p^1, p^2) = H\left(\frac{p^1+p^2}{2}\right) - \frac{H(p^1)+H(p^2)}{2}$. We defined the tissue specificity score of a given gene with expression E in tissue t as $TS(E|E^t) = 1 - \sqrt{JS(E, E^t)}$.

To investigate the cell specificity of HRGs in brain cell types, we downloaded the cell specificity values obtained by single-cell RNAseq analysis from a previous study[11] calculated using the

generate.celltype.data function of the EWCE package and used a one-sided Wilcoxon rank-sum test with Bonferroni correction to compare the cell specificity of HRGs and LBGs. Among 24 cell types, four cell types proved to be as strongly associated with SCZ as in the previous study[11]. Those cell types were hippocampal CA1 pyramidal cells, striatal medium spiny neurons, neocortical somatosensory pyramidal cells, and cortical interneurons.

Datasets used for tissue specific analysis

To evaluate the expression of risk genes in multiple tissues, we collected gene expression data from the Genotype-Tissue Expression project (GTEx) release V6. GTEx includes RNA-seq data from 8,555 samples on 53 human tissues, from which we downloaded 56,318 (RPKM) values of genes in 53 tissues including 13 brain tissues. We used the median RPKM (Reads Per Kilobase of transcript per Million mapped reads) for each tissue in the GTEx data and obtained 318,197 RPKM values for use in this study. Additionally, RNA-seq data from Brain eQTL Almanac (BrainEAC) were also employed to test the tissue specificity of risk genes. BrainEAC was constructed by the UK Brain Expression Consortium and comprises RNA-seq data from 134 European individuals free of neurodegenerative disorders for 10 brain regions. The 10 brain regions are cerebellar cortex (CRBL), frontal cortex (FCTX), occipital cortex (OCTX), temporal cortex (TCTX), hippocampus (HIPP), putamen (PUTM), thalamus (THAL), substantia nigra (SNIG), medulla (MEDU) and intralobular white matter (WHMT). In total, 1,340 brain tissues are represented in BrainEAC. The median RPKM of a gene over all samples in a brain region was considered as the gene expression value of the brain region.

Enrichment analysis on genes expressed differently in SCZ patients

To test the enrichment of high-risk genes differentially expressed in SCZ patients and controls, we used gene expression data of human post-mortem brain tissues from 71 SCZ patients and 70

controls downloaded from GEO with access number 80655[12]. We used R package DESeq2 to perform the differential expression analysis.

Enrichment analysis on genes in relation to developmental stage of brain

To investigate the enrichment of high-risk genes in relation to developmental stage specificity in brain, we downloaded RNA-Seq RPKM values from BrainSpan[13]. BrainSpan is a foundational resource for studying transcriptional mechanisms involved in human brain development. The RPKM values were averaged across samples for each gene. We performed a one-sided Wilcoxon rank-sum test for average of expression values at five developmental stages (fetal, infancy, childhood, adolescence and adulthood). Here we used the $\log_2$(RPKM) as the expression level of genes.

Application of Random walking with Restart algorithm (RWR) in calculating the moving probability of genes

The distance between the gene from locus *l* and the set of risk gene X_{-l} was calculated by RWR through gene-gene interactions. The gene network includes gene function, protein-protein interaction (PPI), and tissue-specific networks. To be same as the previous study[14], the gene function network was computed from the Gene Ontology (GO) database where genes were connected if sharing any GO identity. The weight matrix W_G was calculated as:

$$W_{G_{ij}} = \sum_{\mathbf{B} \in \{\mathbf{B}: \mathbf{g_i} \in \mathbf{B} \text{ and } \mathbf{g_j} \in \mathbf{B}\}} -2\log\left(\frac{M_B}{M_{total}}\right)$$

where M_B is the number of genes annotated with term B, M_{total} is the total number of genes annotated in GO, and the summation was made on all functional terms owned by g_i and g_j. Afterwards, the matrix will be W_G that was normalized by column to obtain the final gene function matrix.

The PPI network were obtained from the BioGRID database[15] that includes protein interactions from 28 experimental systems (Supplementary Materials). The weight matrix W_P was computed as $W_{P_{ij}} = \frac{N_{ij}}{N_j}$, where N_{ij} is the total number of experimental systems supporting the interactions between proteins i and j; N_j is the total number of proteins interacting with protein j.

The tissue-specific networks were obtained from the TissueNet database covering four brain tissues (cerebral cortex, cerebellum, hippocampus, and lateral ventricle)[16]. The weight matrix of protein interactions from TissueNet was denoted as W_T that was defined as $W_{T_{ij}} = \frac{N_{ij}}{N_j}$, where N_{ij} is the number of common proteins interacting with protein i and j, and N_j is the total number of proteins interacting with protein j.

The weighted network was used in RWR to calculate the distance between the gene from locus l and the risk genes. First, we set a restarting rate r, which means the gene either moving to other genes with a probability $1 - r$ or moving back to itself with a probability r. r was set to 0.3 here. Let $t_{l,k}$ be the probability of all genes being reached at step k starting with gene l. The probability for the next step of moving can be formalized as $t_{l,k+1} = (1 - r)Wt_{l,k} + rs_l$, where s_l is a vector with the l-th element as 1 and other elements as 0, and $t_{l,0}$ is set to be the interaction weights of gene l with all other genes in the weight matrix W. This process was iterated until $|t_{l,k+1} - t_{l,k}| < E_{rwr}$, where E_{rwr} is a predefined threshold set as $1e - 6$, and gives t_l, the moving probability of gene l to other genes. t_l were collected into the movement probabilities of candidate genes R. Let $R = [t_1, t_2, \dots, t_k]$, k is the number of genes involved in the network. Above process was performed through three kinds of networks, GO network, BioGRID network, and TissueNet network, respectively. If a pair of genes were found to be interacting in more than one networks, the scores in R calculated by RWR were summed. The conditional probability of gene l contributed by N, $P(X_l | X_{-l}, N)$ can be calculated as the sum of the sub-vectors extracted from R, which are rows for X_l and columns for X_{-l} in R.

Results

Relation of high risk genes predicted by GO and PPI networks with multi-omics data

Integrating the GO network, the BioGRID network and the network from hippocampus predicted 106 HRGs and 828 LBGs. We found that these HRGs were associated with nine gross deletions whereas the LBGs have not been reported to harbour any gross deletions (one-sided Fisher's exact test: $p = 1.43 \times 10^{-3}$). Moreover, the HRGs contained more enhancers in DRE-promoters from CapHiC (one-sided Wilcoxon rank-sum test: $p = 8.28 \times 10^{-4}$, Fig.S1 and Fig.S2a) than the LBGs. When we integrated the gross deletion information and the CapHiC data into rGAT-omics, we observed that the HRGs are more enriched in DNMs, have more DRE-promoters, and are more enriched in differentially expressed genes (DE) than the LRGs (Fig.S2b). We also noted that the HRGs were more likely to exhibit developmental stage specificity in all developmental stages (fetal, infancy, childhood, adolescence and adulthood) than the LBGs according to a stage specificity investigation on BrainSpan data by a one-sided Wilcoxon rank-sum test as shown in Fig.S2c.

Next, we investigated the distance between the HRGs identified by the integrated networks to the index SNPs (DTS). Among the 106 HRGs, 23 (21.7%) were the genes in closest proximity to the corresponding index SNPs, which is significantly higher than would be expected by chance alone ($p < 10^{-6}$, permutation test). This indicated that although the nearest genes to the index SNPs were not necessarily risk genes, distance is still a contributory factor in the prediction.

Involvement of HRGs in biological functions related to SCZ

The biological processes (BP) function enrichment test of the HRGs was performed by g:Profiler[17] on 103 HRGs predicted by rGAT-omics. In all, 45 functions were found to be significantly ($p_{adj} < 0.05$) enriched by the HRGs, which included 12 functions involving neuron or brain activity (Fig.S4; Table S3). These functions included the voltage-gated calcium channel and signaling function, which was found to be significantly enriched in HRGs and to involve

GPM6A and *GRIN2A* specifically. The relationship between this function and SCZ has been previously highlighted by a number of studies[18-20]. Here, *GPM6A* and *GRIN2A* genes were respectively found to capture 213 and 282 links in CapHiC, and they captured many more links than the average value of 74 for all candidate genes. We also identified HRGs enriched in the function of neurogenesis, involving a total of 26 genes shown in Table S3. Among them, *CLU* has already been implicated in SCZ[2]. Another biological function, nervous system development, was recognized as being enriched among the HRGs. This function was exhibited by 38 HRGs that included genes already known to be closely related to neurological disorders (Table S3). For example, *GPM6A* is involved in stress-induced hippocampal alterations in psychiatric disorders[19] whereas abnormal expression of *ZNF804A* has been implicated in increased susceptibility to SCZ[21]. Moreover, function brain development was associated with the HRGs, including specifically the *ATP6V0D1* gene. *ATP6V0D1*, which encodes a component of vacuolar ATPase, has been found to be upregulated in the dorsolateral prefrontal cortex from patients with SCZ by comparison with controls[22]. These results suggested that the HRGs identified by rGAT-omics are genuine SCZ risk genes and potentially reflect underlying pathogenic mechanisms.

Importantly, 23 genes were predicted as HRGs by rGAT-omics but not included in the SCZ-related gene sets (Table S4). Among them, three genes, *BTG1*, *MMP16* and *BRINP2,* have been reported as being expressed significantly differently between SCZ patients and controls. Briefly, the *BTG1* gene has been reported as a blood biomarker of SCZ and has also been found to be differentially expressed in the brains of SCZ patients[23, 24]; the *Mmp16* gene has been reported to show altered expression in brain tissues of a SCZ rat model compared to controls[25]; the *BRINP2* gene has been found to be differentially expressed between the SCZ patient group and healthy controls[26]. Additionally, *SNX8* has been reported as a β-amyloid (Aβ) toxicity enhancer and associated with Alzheimer disease[27]; finally, *CRBN* has been demonstrated to play a role in tau protein accumulation in frontotemporal dementia (FTD)[28].

High involvement of HRGs in targets of nervous system drugs

Drug-Protein binding data were downloaded from BindingDB (www.bindingdb.org/bind/index.jsp) and DrugBank (https://www.drugbank.ca/releases/latest). Data were first filtered by four separate criteria: (1) The protein target is human (*Homo sapiens*); (2) The binding pocket of target protein is constituted by only one chain; (3) The protein target has at least one available Uniprot ID; (4) The ligand-protein binding affinity is less than 10 μM (either one of K_i, K_d, IC_{50} and EC_{50}). The ligands in SMILES format were transformed into InChiKey representing each ligand by OpenBabel[29] to avoid more than one SMILES representing one ligand. This yielded 523,611 pairs of ligand-protein interactions in BindingDB, which comprised 368,313 ligands binding to 1,572 protein targets. Among these ligands, 1,540 have a DrugBank ID. By using DrugBank ID, we queried DrugBank and obtained 15,750 ligand-protein interactions comprising 4,371 ligands binding to 2,438 protein targets. We next combined the interactions from BindingDB and DrugBank, and obtained 537,720 unique ligand-protein interactions. The ligands with ATC code "N" in the first-class were considered to be drugs with nervous system effect.

In total, 2,978 proteins were collated as targets for 368,286 drugs employed in the treatment of diseases of the human nervous system after merging interactions across the two databases. Among them, 24 (23.3%) out of 103 HRGs constitute targets for 4,054 nervous system drugs, which is a significant enrichment compared to LBGs ($p = 1.90 \times 10^{-3}, OR = 2.25$). These results suggest the potential involvement of HRGs in the etiology of SCZ and other neurological diseases.

Eleven of the 24 HRGs were not identified by iRIGS. For example, *FTL* was missed by iRIGS; *FTL* encodes the light subunit of the ferritin protein and is the target of three drugs (Protoporphyrin, Sodium ferric gluconate complex and Ferric pyrophosphate citrate). The *FTL* gene product is known to be associated with a neurodegenerative disorder related to iron accumulation in the brain[30]. *YWHAE*, a target of the drug fusicoccin, plays an important role in diverse biochemical activities related to signal transduction and has been shown to be associated with various brain abnormalities, learning disabilities and seizures[31]; the *PPP3CA* gene, a target of the drug Myristic acid, has been

found to cause a severe neurodevelopmental disease with seizures and childhood neurodevelopmental disorders[32]. The drug targets missed by iRIGS are shown in Table S5.

Novel high risk genes predicted by rGAT-omics and related with neurological disorders

rGAT-omics predicted 16 genes (Table S4) as novel candidates associated with SCZ, including ten genes reported as being associated with neurological disorders. The novel SCZ gene *SNX8* has been reported to be a β-amyloid (Aβ) toxicity enhancer associated with Alzheimer disease[33]. The overexpression of *SNX8* has been found to exacerbate aberrant handling of neuronal cholesterol[27]. A missense mutation in *NDUFAF4* has been found to cause Leigh syndrome, a severe neurological disorder[34]. Many other studies have shown that *NDUFAF4* is related to mitochondrial disorders[35-37]. An exome sequencing study of 152 families has revealed that *LRRIQ3* is associated with a neurodevelopmental disorder[38]. Recently, an association has been reported between a SNP within *LRRIQ3* and major depression disorder[39]. *MATN1* is reported to associate with ischemic stroke[40]. A gene-based association study[41] has recently revealed an association between *MATN1* and Adolescent idiopathic scoliosis. Mutations in *CRBN* have been implicated as a cause of intellectual disability[42, 43]. *VPS37B* is a gene regulated by glucocorticoids in brain, and is involved in vesicular transport and endosomal sorting complex[44]. The *FTL* gene is involved in inflammation and neurodegenerative disease, and has been reported to be associated with Alzheimer disease[45]. Mutations in the *FTL* gene can cause neuroferritinopathies with brain iron accumulation[46]. Copy number variations involving the *BRINP2* gene have been reported to be associated with neurodevelopmental disorders (NDDs)[47]. *BRINP2* has also been linked to substance abuse disorder and reward dependence in two GWAS[48, 49]. *STAU1* is a key factor in neuronal differentiation, and plays a role in post-transcriptional regulation[50], and is also important for synaptic function[51, 52]. The *NCAPD3* gene has been shown to be associated with microcephaly[53] and neurodevelopmental

syndrome[54]. Thus, we deem it worthwhile to investigate the functions of these genes in SCZ development.

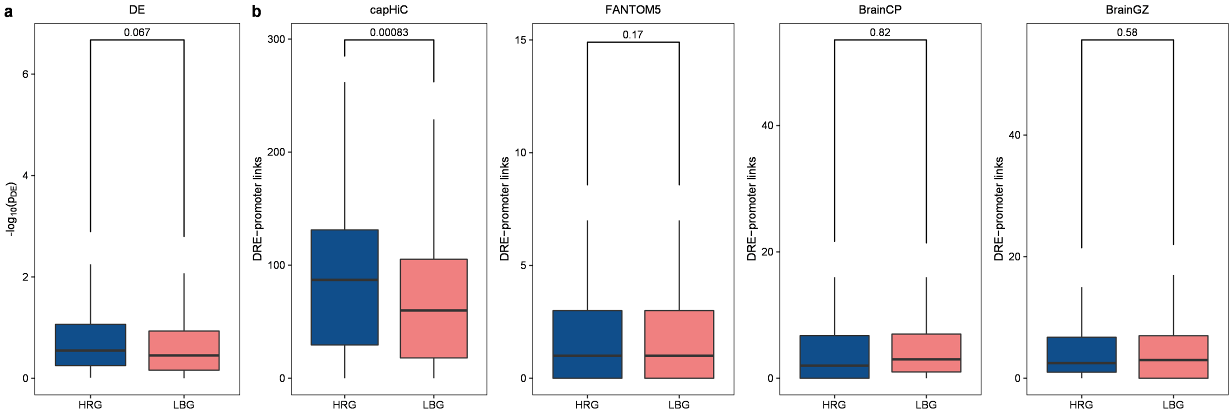

Fig.S1 Genomic features of the HRGs predicted by rGAT-net. (a) HRGs are not more likely to exhibit differentially expressed genes than LBGs in SCZ patients. (b) HRGs harbored significantly more DRE-promoter links in CapHiC data whereas significance disappeared in other sources of promoter links.

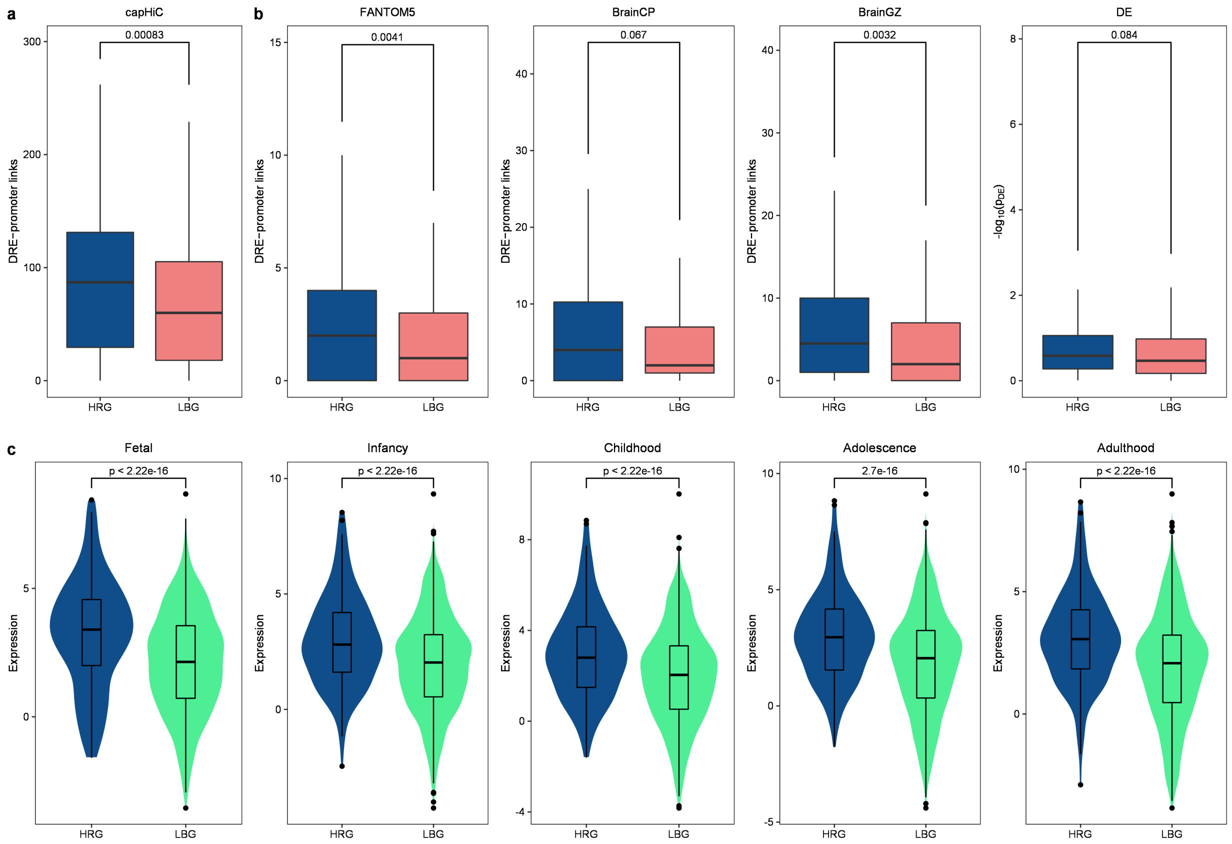

Fig.S2 Genomic features of the HRGs predicted by integrated networks. (a) HRGs (n=106) predicted by GO and PPI networks capture more DRE-promoter links in CapHiC data compared to LBGs (n=828). (b) HRGs (n=104) predicted by integrating CapHiC and gross deletion information with networks capture more DRE-promoters in FANTOM5, BrainCP and BrainGZ data. Moreover, HRGs are more likely to exhibit differential expression compared to LBGs. (c) HRGs identified by rGAT-net are highly expressed in all developmental stages (fetal, infancy, childhood, adolescence and adulthood) in BrainSpan data.

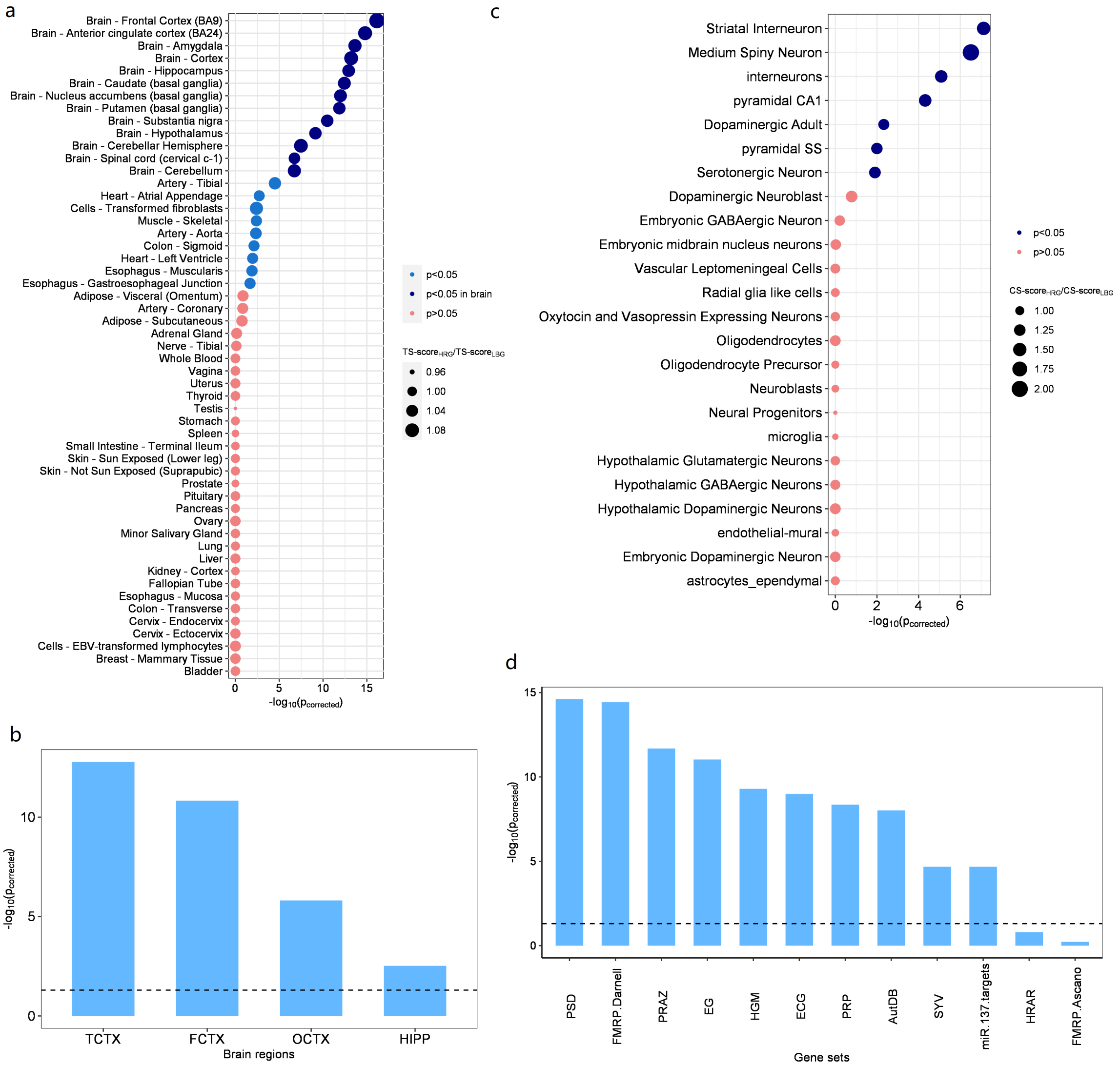

Fig.S3 Comparison of HRGs and LBGs identified by rGAT-omics. (a) HRGs (n=103) were highly expressed in 13 brain tissues compared to LBGs (n=849). The size of the circles is based on the ratio of the median tissue-specificity scores of HRGs and LBGs in each tissue. (b) HRGs were highly expressed in four brain regions in BrainEAC data. (c) HRGs were specifically expressed in seven cell types in brain and of these, four were strongly associated with SCZ. The size of the circles represents the ratio of median cell-specificity values of HRGs and LBGs in each cell type. (d) HRGs were enriched in 10 SCZ-related gene sets compared to LBGs.

GO:BP		stats	
Term na...	Term ...	p...	0 −log10(p... ≤16
cellular component organization	GO:0016043	5.438×10^{-9}	
cellular component organization or biogenesis	GO:0071840	6.131×10^{-9}	
anatomical structure development	GO:0048856	2.379×10^{-7}	
nervous system development	GO:0007399	5.348×10^{-7}	
system development	GO:0048731	9.697×10^{-7}	
multicellular organism development	GO:0007275	2.590×10^{-6}	
developmental process	GO:0032502	3.618×10^{-6}	
head development	GO:0060322	3.158×10^{-5}	
regulation of biological quality	GO:0065008	4.035×10^{-5}	
brain development	GO:0007420	6.849×10^{-5}	
cellular developmental process	GO:0048869	8.060×10^{-5}	
multicellular organismal process	GO:0032501	1.323×10^{-4}	
synapse organization	GO:0050808	2.349×10^{-4}	
anatomical structure morphogenesis	GO:0009653	4.362×10^{-4}	
cell differentiation	GO:0030154	4.962×10^{-4}	
neurogenesis	GO:0022008	6.982×10^{-4}	
negative regulation of cellular process	GO:0048523	7.938×10^{-4}	
central nervous system development	GO:0007417	1.850×10^{-3}	
negative regulation of nitrogen compound metabolic process	GO:0051172	2.858×10^{-3}	
cell-cell signaling	GO:0007267	3.125×10^{-3}	
positive regulation of gene expression	GO:0010628	3.220×10^{-3}	
negative regulation of biological process	GO:0048519	3.230×10^{-3}	
positive regulation of biological process	GO:0048518	3.997×10^{-3}	
homeostatic process	GO:0042592	4.255×10^{-3}	
positive regulation of multicellular organismal process	GO:0051240	4.786×10^{-3}	
animal organ development	GO:0048513	5.872×10^{-3}	
generation of neurons	GO:0048699	9.257×10^{-3}	
regulation of cellular component organization	GO:0051128	9.989×10^{-3}	
regulation of cell communication	GO:0010646	1.202×10^{-2}	
biological regulation	GO:0065007	1.226×10^{-2}	
positive regulation of cellular process	GO:0048522	1.358×10^{-2}	
cellular component biogenesis	GO:0044085	1.369×10^{-2}	
regulation of synapse structure or activity	GO:0050803	1.485×10^{-2}	
regulation of signaling	GO:0023051	1.595×10^{-2}	
cellular component assembly	GO:0022607	1.700×10^{-2}	
neuron differentiation	GO:0030182	1.726×10^{-2}	
modulation of chemical synaptic transmission	GO:0050804	1.763×10^{-2}	
regulation of trans-synaptic signaling	GO:0099177	1.803×10^{-2}	
negative regulation of macromolecule metabolic process	GO:0010605	2.445×10^{-2}	
chemical homeostasis	GO:0048878	2.461×10^{-2}	
regulation of multicellular organismal process	GO:0051239	3.098×10^{-2}	
synaptic signaling	GO:0099536	3.197×10^{-2}	
cellular chemical homeostasis	GO:0055082	3.478×10^{-2}	
negative regulation of cellular metabolic process	GO:0031324	4.083×10^{-2}	
cell development	GO:0048468	4.472×10^{-2}	

Fig.S4 Function enrichment analysis on HRGs by GO biological process. HRGs were found to be enriched in 45 biological processes in g:Profiler.

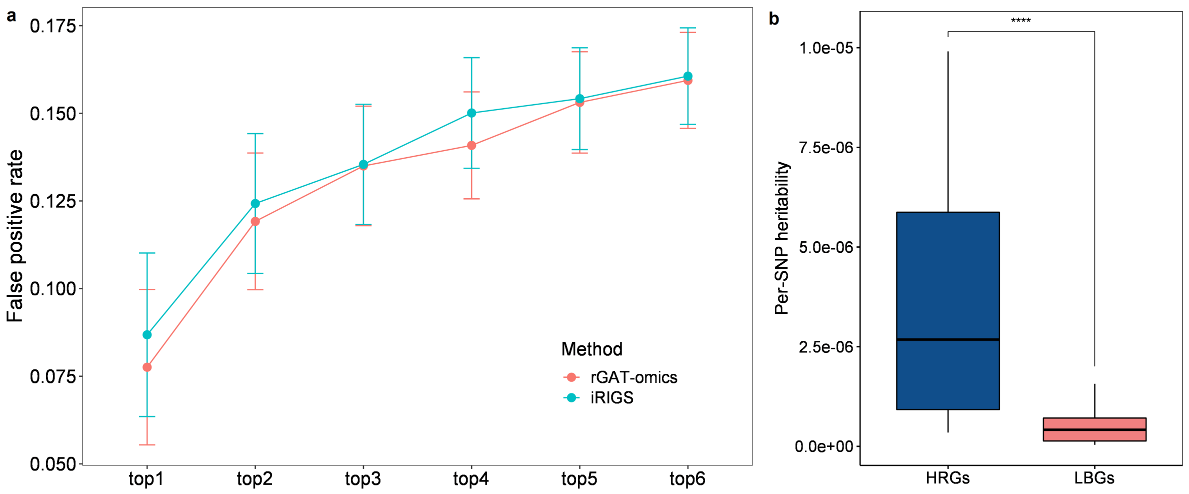

Fig.S5 Evaluation of rGAT-omics on false positive rate and per-SNP heritability. (a) The false positive rate (FPR) of rGAT-omics and iRIGS for genes predicted as the top1 to top6 associations of SCZ SNPs. Each spot denotes the mean value of FPRs, and the error bar means the standard deviation of that set of FPRs. The FPRs of rGAT-omics were significantly lower than the FPRs of iRIGS base on one-sided Wilcoxon rank-sum test. (b) The average heritability of SNPs around HRGs is significantly higher than the average heritability of SNPs around LBGs according to one-sided Wilcoxon rank-sum test.

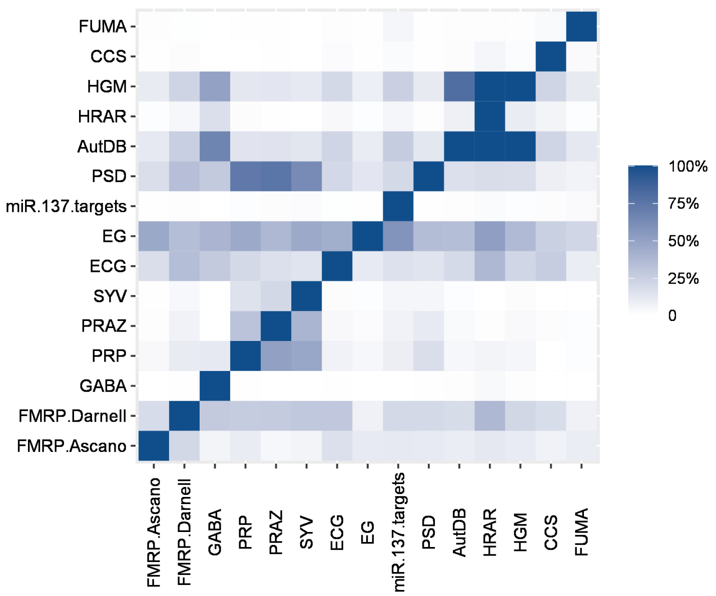

Fig.S6 The proportions of overlapping genes between 15 SCZ-related gene sets.

Table S1 Gene set enrichment of the non-nearest genes using the nearest genes as background.

Gene set	$p_{corrected}$	OR
**PSD**	**1.02×10^-5^**	17.79
**PRP**	**2.31×10^-3^**	∞
**PRAZ**	**5.05×10^-3^**	∞
FMRP.Darnell	0.12	3.33
SYV	0.22	∞
EG	0.23	2.40
AutDB	1	0.53
CCS	1	0
ECG	1	1.09
FMRP.Ascano	1	1.80
FUMA	1	0.04
GABA	1	0
HGM	1	0.63
HRAR	1	0.98
miR.137.targets	1	∞

∞ denotes no LBGs were involved in that gene set; thus, OR turns infinite.

Table S2 Gene sets used for gene enrichment analysis.

Gene set	# of genes	Descriptions of the gene set
AutDB[*]	1141	Genes related to autism from AutDB[55]
CCS[**]	73	Voltage-gated calcium-channel subunits[56]
ECG[*]	1003	Evolutionarily constrained genes[57]
EG[*]	3915	Essential genes[58]
FMRP.Ascano[**]	939	FMRP targets[59]
FMRP.Darnell[**]	842	FMRP targets[60]
FUMA[***]	84	Risk genes of SCZ predicted by FUMA[61]
GABA[***]	18	$GABA_A$ receptor complex[62]
HGM[*]	913	Genes related with autism from Human Gene Module[63]
HRAR[*]	91	Highest highest-ranking autism risk genes[63]
miR.137.targets[***]	45	Targets of miR-137[64]
PRAZ[**]	209	Genes related to presynaptic active zone[65]
PRP[**]	335	Genes related to presynaptic proteins[65]
PSD[**]	1447	Genes associated with postsynaptic density[66]
SYV[**]	107	Genes related to synaptic vesicles[65]

[*] Gene set including genes associated with autism

[**] Gene set including genes involving in synaptic, presynaptic, or voltage-gated calcium-channel functions

[***] Gene set including genes associated with SCZ.

Table S3 HRGs enriched in neurological functions.

Description of biological process	# of HRGs	Gene symbol
Neurogenesis	26	*KIF5C, SATB2, PALLD, GPM6A, ZNF804A, NDRG4, NLGN4X, RORA, MEF2C, ZNF536, PRKD1, NCAM1, PPP3CA, PTN, YWHAE, NGRN, TMEFF2, BRINP2, CAPRIN2, CHD7, TCF4, CLU, PLXNA2, BTBD1, SOX2, GRIN2A*
Head development	20	*SATB2, INA, NDRG4, DDX10, NLGN4X, CKB, RORA, PPARGC1A, PPP3CA, PTN, YWHAE, NRGN, XRCC6, CHD7, PLXNA2, AKT3, ATP6V0D1, CTNND1, SOX2, GRIN2A*
Brain development	19	*AKT3, ATP6V0D1, CHD7, CKB, CTNND1, GRIN2A, INA, NDRG4, NLGN4X, NRGN, PLXNA2, PPARGC1A, PPP3CA, PTN, RORA, SATB2, SOX2, XRCC6, YWHAE*
Synapse organization	14	*GPM6A, INA, ZNF804A, NLGN4X, LRRN3, CNKSR2, MEF2C, SYBU, PTN, BAI1, TMEFF2, CAPRIN2, STAU1, HSPA8*
Nervous system development	38	*KIF5C, SATB2, PALLD, GPM6A, INA, ZNF804A, NDRG4, NLGN4X, CKB, RORA, LRRN3, MEF2C, ZNF536, PRKD1, PPARGC1A, DPF3, NCAM1, FKBP8, PPP3CA, SYBU, PTN, YWHAE, NGRN, NRGN, TMEFF2, XRCC6, BRINP2, CAPRIN2, CHD7, TCF4, CLU, PLXNA2, AKT3, ATP6V0D1, CTNND1, BTBD1, SOX2, GRIN2A*
Central nervous system development	20	*SATB2, INA, NDRG4, NLGN4X, CKB, RORA, PPARGC1A, PPP3CA, PTN, YWHAE, NRGN, XRCC6, CHD7, CLU, PLXNA2, AKT3, ATP6V0D1, CTNND1, SOX2, GRIN2A*
Regulation of synapse structure or activity	9	*GPM6A, ZNF804A, LRRN3, MEF2C, SYBU, PTN, BAI1, CAPRIN2, HSPA8*
Neuron differentiation	22	*KIF5C, SATB2, PALLD, GPM6A, ZNF804A, NDRG4, NLGN4X, RORA, MEF2C, ZNF536, PRKD1, NCAM1, PPP3CA, PTN, BAI1, NGRN, TMEFF2, BRINP2, CAPRIN2, TCF4, PLXNA2, SOX2*
Regulation of trans-synaptic signaling	12	*GRIA1, GRM3, NLGN4X, MEF2C, PPP3CA, SYBU, PTN, BAI1, ATP2A2, NRGN, STAU1, GRIN2A*
Modulation of chemical synaptic transmission	12	*GRIA1, GRM3, NLGN4X, MEF2C, PPP3CA, SYBU, PTN, BAI1, ATP2A2, NRGN, STAU1, GRIN2A*
Generation of neurons	23	*BAI1, BRINP2, CAPRIN2, CHD7, GPM6A, KIF5C, MEF2C, NCAM1, NDRG4, NGRN, NLGN4X, PALLD, PLXNA2, PPP3CA, PRKD1, PTN, RORA, SATB2, SOX2, TCF4, YWHAE, ZNF536, ZNF804A*
Synaptic signaling	15	*GRIA1, GRM3, NLGN4X, SV2A, MEF2C, PPP3CA, SYBU, PTN, BAI1, GNB1, ATP2A2, NRGN, STAU1, HSPA8, GRIN2A*

Table S4 Novel genes predicted by rGAT-omics but missed by other methods.

Novel genes missed by other methods	# of HRGs	Gene symbol
HRGs missed by SCZ-related gene sets	23	*SNX8, LUZP2, FANCL, NDUFAF4, LRRIQ3, LRRN3, MATN1, CRBN, DPF3, VPS37B, BTG1, FTL, PJA1, XRCC6, RPRD1B, BRINP2, CAPRIN2, STAU1, NCAPD3, MLH1, MMP16, STAM, BTBD1*
dHRGs missed by SCZ-related gene sets	17	*SNX8, FANCL, NDUFAF4, LRRIQ3, MATN1, CRBN, DPF3, VPS37B, FTL, XRCC6, RPRD1B, BRINP2, STAU1, NCAPD3, MLH1, STAM, BTBD1*
HRGs missed by all other methods	16	*SNX8, NDUFAF4, LRRIQ3, MATN1, CRBN, DPF3, VPS37B, FTL, XRCC6, RPRD1B, BRINP2, STAU1, NCAPD3, MLH1, STAM, BTBD1*

Table S5Targets of nervous system drugs predicted by rGAT-omics but missed by iRIGS.

Target (UniProt ID)	Gene symbol	DrugBank ID
P06733	*ENO1*	DB11638
Q9UBS5	*GABBR1*	DB00181, DB00837, DB00996, DB01080, DB01956, DB02530, DB05010, DB06354, DB08891, DB08892
P04075	*ALDOA*	DB02512, DB04326, DB04733, DB08240, DB11638
P06576	*ATP5B*	DB04216, DB07384, DB07394, DB08399
P12277	*CKB*	DB00148, DB13191
Q7L0J3	*SV2A*	DB01202, DB05541, DB05885
Q96SW2	*CRBN*	DB00480, DB01041, DB08910
Q08209	*PPP3CA*	DB00864, DB00864
P62258	*YWHAE*	DB01780
P02792	*FTL*	DB02285
P78362	*SRPK2*	DB01268, DB08568, DB00173, DB02733, DB04395
P06733	*ENO1*	DB11638

Table S6 Gene set enrichment of risk genes missed by Maha-integration and rGAT-omics.

	**rGAT-omics**[a]		**Maha-integration** [b]	
**Gene set**	$p_{corrected}$	**OR**	$p_{corrected}$	**OR**
PSD	**4.90×10^{-3}**	11.69	0.29	6.50
EG	**0.017**	5.61	**1.53×10^{-3}**	8.10
FMRP.Darnell	0.30	6.31	1	4.21
PRP	0.61	7.63	1	0
HRAR	0.70	42.67	1	0
AutDB	1	3.93	0.26	6.79
CCS	1	0	1	15.35
ECG	1	3.47	0.35	5.98
FMRP.Ascano	1	1.51	1	3.44
FUMA	1	1.94	1	0
GABA	1	0	1	0
HGM	1	5.06	0.14	8.72
miR.137.targets	1	0	1	0
PRAZ	1	0	1	0
SYV	1	21.52	1	0

[a] The enrichment P-values and Odds Ratio (OR) of HRGs predicted by rGAT-omics but missed by Maha-integration; [b] The enrichment P-values and ORs of HRGs predicted by Maha-integration but missed by rGAT-omics. Background genes used here were common LBGs predicted by both rGAT-omics and Maha-integration.

Table S7 Number of HRGs and LBGs identified by rGAT-net.

Network	HRGs	LBGs
GO	105	842
BioGRID[a]	99	466
CCTX[b]	102	467
CRBL[c]	101	399
HIPP[d]	100	379
LTVC[e]	99	384
GO+BioGRID+CCTX	106	847
GO+BioGRID+CRBL	106	847
GO+BioGRID+HIPP	106	828
GO+BioGRID+LTVC	106	842

[a] BioGRID, [b] CCTX, [c] CRBL, [d] HIPP and [e] LTVC represent the PPI networks derived from the BioGRID database and TissueNet database for four types of tissue including cerebral cortex (CCTX), cerebellum (CRBL), hippocampus (HIPP), and lateral ventricle (LTVC) respectively. "+" represents integrating networks.

Table S8 Tissue-specificity evaluation on HRGs predicted by GO network.

**Brain-related tissue**[a]	**GO network**[b]
Brain - Spinal cord (cervical c-1)	0.075
Brain – Amygdala	0.25
Brain - Substantia nigra	0.27
Brain - Frontal cortex (BA9)	0.50
Brain – Hippocampus	0.55
Brain - Anterior cingulate cortex (BA24)	0.87
Brain - Caudate (basal ganglia)	1
Brain - Cerebellar hemisphere	1
Brain – Cerebellum	1
Brain – Cortex	1
Brain – Hypothalamus	1
Brain - Nucleus accumbens (basal ganglia)	1
Brain - Putamen (basal ganglia)	1
**Brain region**[c]	**GO network**
**TCTX**	**0.024**
FCTX	0.25
HIPP	0.28
MEDU	1
OCTX	1
PUTM	1
SNIG	1
CRBL	1
THAL	1
WHMT	1

[a] Brain-related tissues are extracted from GTEx data; [b] The enrichment P-values of HRGs predicted by rGAT-net using only GO network; [c] Abbreviations of brain regions from BrainEAC.

Table S9 Novel genes interacted with non-novel genes

Network	Novel genes	Non-novel genes
GO	*LRRIQ3, MATN1, CRBN, DPF3, VPS37B, FTL, XRCC6, BRINP2, STAU1*	*ENO1, CKAP5, KIF5C, SATB2, TNRC6B, ZMAT2, SNAP91, MPP6, PALLD, APLP2, IGF1, GPM6A, GRIA1, TLE1, YBX1, GABBR1, FANCL, CUL3, HAND1, TLE3, NDRG4, ALDOA, NLGN4X, CKB, RORA, KCNQ5, CNKSR2, MEF2C, DPYD, ERC1, PRKD1, PPARGC1A, GNL3, YY1, FKBP8, DPP4, PPP3CA, SYBU, PTN, HSPA9, BTG1, SATB1, PJA2, PDE4D, YWHAE, GNB1, NGRN, ATP2A2, THBS1, PJA1, TMEFF2, SF3B1, SRPK2, ATG16L1, CAPRIN2, CHD7, TCF4, CLU, ERCC4, STAG1, PLXNA2, AKT3, HSPA8, MKLN1, MORF4L1, ATP6V0D1, COPS3, PSMD6, CTNND1, SOX2, GRIN2A, LUZP2, DDX10, SV2A*
BioGRID	*FTL, MLH1, STAU1, XRCC6, CRBN*	*PTN, CKB, ALDOA, FKBP8, PSMD6, ATP6V0D1, HSPA9, INA, ATP5B, YBX1, ENO1, HSPA8, SF3B1, YY1, CLU*
TissueNet (hippocampus)	*SNX8, CRBN, DPF3, XRCC6, STAU1, MLH1, STAM, BTBD1*	*ENO1, TNRC6B, YBX1, CUL3, HSPA9, BTG1, ATG16L1, HSPA8, COPS3, CHD7, LUZP2, INA, DDX10, ALDOA, CKB, LRRN3, GNL3, KIF5C, CYP26B1*

Table S10 Candidate genes with PP ranked in the top 10% predicted by rGAT-omics.

Index SNP	Gene symbol
chr1_8424984_D	*ENO1, PARK7*
chr11_46350213_D	*CKAP5, DGKZ, MADD*
chr2_149429178_D	*KIF5C*
chr2_200825237_I	*SATB2, SPATS2L*
chr22_39987017_D	*TNRC6B, RPL3, ATF4, SUN2*
chr5_140143664_I	*ZMAT2, DIAPH1, NDUFA2*
chr6_84280274_D	*SNAP91*
chr7_2025096_I	*SNX8, NUDT1, INTS1*
chr7_24747494_D	*MPP6*
rs10503253	*CSMD1*
rs10520163	*PALLD*
rs10791097	*APLP2, NTM*
rs10860964	*IGF1, HSP90B1*
rs11027857	*LUZP2*
rs1106568	*GPM6A*
rs111294930	*GRIA1*
rs11139497	*TLE1*
rs11191419	*INA, ARL3, ACTR1A, PDCD11*
rs11210892	*YBX1, PPIH, MED8, EBNA1BP2, ST3GAL3, IPO13*
rs115329265	*GABBR1, HIST1H2BM, TRIM27, ZNF165, HIST1H2BL, HIST1H2BO*
rs11682175	*FANCL*
rs11685299	*CUL3*
rs11693094	*ZNF804A*
rs117074560	*NDUFAF4*
rs11740474	*HAND1, LARP1*
rs12129573	*LRRIQ3*
rs12148337	*TLE3*
rs12325245	*NDRG4, GOT2*
rs12421382	*DDX10*
rs12691307	*ALDOA, MAZ, MAPK3, SEZ6L2, SRCAP, RNF40, CDIPT, KCTD13, TMEM219, PPP4C, YPEL3, CORO1A, TBC1D10B, ZNF747, ZNF629*
rs12704290	*GRM3*
rs12826178	*ATP5B, KIF5A, LRP1, STAT6, DCTN2, DTX3, CTDSP2*
rs12845396	*NLGN4X*
rs12887734	*CKB, KLC1, EIF5*
rs12903146	*RORA*
rs13240464	*LRRN3*
rs1339227	*KCNQ5*
rs1378559	*CNKSR2*
rs140505938	*SV2A, PRUNE, VPS45, HIST2H2AB, ADAMTSL4*
rs1498232	*MATN1*
rs1501357	*MRPS30*
rs16867576	*MEF2C*
rs1702294	*DPYD*

rs17194490	*CRBN*
rs2007044	*ERC1, FKBP4*
rs2053079	*ZNF536*
rs2068012	*PRKD1*
rs211829	*LRRN3*
rs215411	*PPARGC1A*
rs2332700	*DPF3*
rs2514218	*NCAM1, DRD2*
rs2535627	*GNL3, PBRM1, WDR82, NISCH, ALAS1*
rs2693698	*YY1, BCL11B*
rs2851447	*VPS37B, SBNO1, DIABLO, RSRC2*
rs2905426	*FKBP8, UPF1, TMEM59L, UBA52, COPE, GATAD2A*
rs2909457	*DPP4*
rs35518360	*PPP3CA, UBE2D3*
rs36068923	*SYBU*
rs3735025	*PTN*
rs3768644	*CYP26B1*
rs3849046	*HSPA9, MATR3, PAIP2*
rs4129585	*BAI1, LYNX1, SLC45A4*
rs4240748	*BTG1, EEA1*
rs4330281	*SATB1*
rs4388249	*PJA2*
rs4391122	*PDE4D*
rs4523957	*YWHAE, TSR1, PAFAH1B1, RPA1*
rs4648845	*GNB1, GABRD, SKI, MIB2, C1orf86*
rs4702	*NGRN, FES, UNC45A*
rs4766428	*ATP2A2, ANAPC7, ARPC3*
rs55661361	*NRGN, ROBO3, SIAE, FEZ1, ROBO4*
rs56205728	*THBS1, SRP14, FSIP1, PLCB2*
rs56873913	*FTL, RPS11, SLC17A7, PRMT1, AP2A1, CA11, PIH1D1, IRF3, SNRNP70, RPL13A, RRAS*
rs5937157	*PJA1*
rs59979824	*TMEFF2*
rs6002655	*XRCC6, NHP2L1, SEPT3, TCF20, RANGAP1*
rs6065094	*RPRD1B, TTI1*
rs6434928	*SF3B1, HECW2*
rs6466055	*SRPK2*
rs6670165	*BRINP2*
rs6704641	*SATB2*
rs6704768	*ATG16L1, GIGYF2, DGKD*
rs679087	*CAPRIN2*
rs6984242	*CHD7*
rs7267348	*STAU1, RNF114*
rs72934570	*TCF4*
rs73229090	*CLU, DPYSL2*
rs7405404	*ERCC4*

rs7432375	*STAG1*
rs75059851	*NCAPD3, B3GAT1*
rs7523273	*PLXNA2, FAIM3*
rs75968099	*MLH1*
rs77149735	*AKT3*
rs77502336	*HSPA8, SCN3B, UBASH3B, GRAMD1B, TMEM225*
rs7801375	*MKLN1*
rs7819570	*MMP16*
rs7893279	*STAM*
rs8042374	*MORF4L1, DNAJA4, IREB2*
rs8044995	*ATP6V0D1, PARD6A, CENPT, ACD, PRMT7, EDC4*
rs8082590	*COPS3, RAI1, FLCN, MPRIP*
rs832187	*PSMD6*
rs9420	*CTNND1, SSRP1, TNKS1BP1, ZDHHC5, MED19*
rs950169	*BTBD1, ZNF592*
rs9607782	*TNRC6B, EP300, ST13, XRCC6*
rs9636107	*TCF4*
rs9841616	*SOX2*
rs9922678	*GRIN2A*

* Totally 239 unique genes were with PP ranked in the top 10% and predicted as associated with107 SNPs.

Reference

1. Hoffman, G.E. *et al.* CommonMind Consortium provides transcriptomic and epigenomic data for Schizophrenia and Bipolar Disorder. *Sci Data* **6**, 180 (2019).
2. Won, H. *et al.* Chromosome conformation elucidates regulatory relationships in developing human brain. *Nature* **538**, 523-527 (2016).
3. Mifsud, B. *et al.* Mapping long-range promoter contacts in human cells with high-resolution capture Hi-C. *Nat Genet* **47**, 598-606 (2015).
4. Andersson, R. *et al.* An atlas of active enhancers across human cell types and tissues. *Nature* **507**, 455-461 (2014).
5. Fromer, M. *et al.* De novo mutations in schizophrenia implicate synaptic networks. *Nature* **506**, 179-184 (2014).
6. Gulsuner, S. *et al.* Spatial and temporal mapping of de novo mutations in schizophrenia to a fetal prefrontal cortical network. *Cell* **154**, 518-529 (2013).
7. Girard, S.L. *et al.* Increased exonic de novo mutation rate in individuals with schizophrenia. *Nat Genet* **43**, 860-863 (2011).
8. Xu, B. *et al.* De novo gene mutations highlight patterns of genetic and neural complexity in schizophrenia. *Nat Genet* **44**, 1365-1369 (2012).
9. Stenson, P.D. *et al.* The Human Gene Mutation Database (HGMD((R))): optimizing its use in a clinical diagnostic or research setting. *Hum Genet* (2020).
10. Cabili, M.N. *et al.* Integrative annotation of human large intergenic noncoding RNAs reveals global properties and specific subclasses. *Genes Dev* **25**, 1915-1927 (2011).
11. Skene, N.G. *et al.* Genetic identification of brain cell types underlying schizophrenia. *Nat Genet* **50**, 825-833 (2018).
12. Ramaker, R.C. *et al.* Post-mortem molecular profiling of three psychiatric disorders. *Genome Med* **9**, 72 (2017).

13. Miller, J.A. *et al.* Transcriptional landscape of the prenatal human brain. *Nature* **508**, 199-206 (2014).
14. Wang, Q. *et al.* A Bayesian framework that integrates multi-omics data and gene networks predicts risk genes from schizophrenia GWAS data. *Nat Neurosci* **22**, 691-699 (2019).
15. Stark, C. *et al.* BioGRID: a general repository for interaction datasets. *Nucleic Acids Res* **34**, D535-539 (2006).
16. Basha, O. *et al.* The TissueNet v.2 database: A quantitative view of protein-protein interactions across human tissues. *Nucleic Acids Res* **45**, D427-D431 (2017).
17. Raudvere, U. *et al.* g:Profiler: a web server for functional enrichment analysis and conversions of gene lists (2019 update). *Nucleic Acids Res* **47**, W191-W198 (2019).
18. Zhang, Z. *et al.* The effects of CACNA1C gene polymorphism on prefrontal cortex in both schizophrenia patients and healthy controls. *Schizophr Res* **204**, 193-200 (2019).
19. Boks, M.P. *et al.* Do mood symptoms subdivide the schizophrenia phenotype? Association of the GMP6A gene with a depression subgroup. *American journal of medical genetics. Part B, Neuropsychiatric genetics : the official publication of the International Society of Psychiatric Genetics* **147B**, 707–711 (2008).
20. Jha, S., Read, S., Hurd, P. & Crespi, B. Segregating polymorphism in the NMDA receptor gene GRIN2A, schizotypy, and mental rotation among healthy individuals. *Neuropsychologia* **117**, 347-351 (2018).
21. Baek, J.H. *et al.* Association between the zinc finger protein 804A (ZNF804A) gene and the risk of schizophrenia and bipolar I disorder across diagnostic boundaries. *Bipolar disorders* **19**, 305–313 (2017).
22. Martins-de-Souza, D. *et al.* Prefrontal cortex shotgun proteome analysis reveals altered calcium homeostasis and immune system imbalance in schizophrenia. *European archives of psychiatry and clinical neuroscience* **259**, 151–163 (2009).
23. Glatt, S.J. *et al.* Comparative gene expression analysis of blood and brain provides concurrent

validation of SELENBP1 up-regulation in schizophrenia. *Proceedings of the National Academy of Sciences of the United States of America* **102**, 15533–15538 (2005).

24. Mohammadi, A., Rashidi, E. & Amooeian, V.G. Brain, blood, cerebrospinal fluid, and serum biomarkers in schizophrenia. *Psychiatry Res* **265**, 25-38 (2018).
25. Paparelli, A. *et al.* Perinatal Asphyxia in Rat Alters Expression of Novel Schizophrenia Risk Genes. *Front Mol Neurosci* **10**, 341 (2017).
26. Zhang, Y. *et al.* Peripheral Blood Leukocyte RNA-Seq Identifies a Set of Genes Related to Abnormal Psychomotor Behavior Characteristics in Patients with Schizophrenia. *Med Sci Monit* **26**, e922426 (2020).
27. Muirhead, G. & Dev, K.K. The expression of neuronal sorting nexin 8 (SNX8) exacerbates abnormal cholesterol levels. *Journal of molecular neuroscience : MN* **53** (2014).
28. Silva, M.C. *et al.* Targeted degradation of aberrant tau in frontotemporal dementia patient-derived neuronal cell models. *Elife* **8** (2019).
29. O'Boyle, N.M. *et al.* Open Babel: An open chemical toolbox. *J Cheminform* **3**, 33 (2011).
30. Maciel, P. *et al.* Neuroferritinopathy: missense mutation in FTL causing early-onset bilateral pallidal involvement. *Neurology* **65**, 603-605 (2005).
31. Noor, A., Bogatan, S., Watkins, N., Meschino, W.S. & Stavropoulos, D.J. Disruption of YWHAE gene at 17p13.3 causes learning disabilities and brain abnormalities. *Clinical genetics* **93**, 365–367 (2018).
32. Myers, C.T. *et al.* De Novo Mutations in PPP3CA Cause Severe Neurodevelopmental Disease with Seizures. *Am J Hum Genet* **101**, 516-524 (2017).
33. Di Paolo, G. & Kim, T.W. Linking lipids to Alzheimer's disease: cholesterol and beyond. *Nat Rev Neurosci* **12**, 284-296 (2011).
34. Baertling, F. *et al.* NDUFAF4 variants are associated with Leigh syndrome and cause a specific mitochondrial complex I assembly defect. *Eur J Hum Genet* **25**, 1273-1277 (2017).
35. Dieteren, C.E. *et al.* Defective mitochondrial translation differently affects the live cell

dynamics of complex I subunits. *Biochim Biophys Acta* **1807**, 1624-1633 (2011).

36. Zurita Rendon, O. & Shoubridge, E.A. Early complex I assembly defects result in rapid turnover of the ND1 subunit. *Hum Mol Genet* **21**, 3815-3824 (2012).
37. Saada, A. *et al.* Mutations in NDUFAF3 (C3ORF60), encoding an NDUFAF4 (C6ORF66)-interacting complex I assembly protein, cause fatal neonatal mitochondrial disease. *Am J Hum Genet* **84**, 718-727 (2009).
38. Reuter, M.S. *et al.* Diagnostic Yield and Novel Candidate Genes by Exome Sequencing in 152 Consanguineous Families With Neurodevelopmental Disorders. *JAMA Psychiatry* **74**, 293-299 (2017).
39. Johnston, K.J.A. *et al.* Identification of novel common variants associated with chronic pain using conditional false discovery rate analysis with major depressive disorder and assessment of pleiotropic effects of LRFN5. *Transl Psychiatry* **9**, 310 (2019).
40. He, W. *et al.* Altered Long Non-Coding RNA Transcriptomic Profiles in Ischemic Stroke. *Human gene therapy* **29**, 719–732 (2018).
41. Chen, Z. *et al.* Promoter polymorphism of matrilin-1 gene predisposes to adolescent idiopathic scoliosis in a Chinese population. *Eur J Hum Genet* **17**, 525-532 (2009).
42. Rajadhyaksha, A.M. *et al.* Behavioral characterization of cereblon forebrain-specific conditional null mice: a model for human non-syndromic intellectual disability. *Behav Brain Res* **226**, 428-434 (2012).
43. Song, T. *et al.* CRL4 antagonizes SCFFbxo7-mediated turnover of cereblon and BK channel to regulate learning and memory. *PLoS Genet* **14**, e1007165 (2018).
44. Juszczak, G.R. & Stankiewicz, A.M. Glucocorticoids, genes and brain function. *Prog Neuropsychopharmacol Biol Psychiatry* **82**, 136-168 (2018).
45. Crespo, A.C. *et al.* Genetic and biochemical markers in patients with Alzheimer's disease support a concerted systemic iron homeostasis dysregulation. *Neurobiol Aging* **35**, 777-785 (2014).

46. Maccarinelli, F. *et al.* A novel neuroferritinopathy mouse model (FTL 498InsTC) shows progressive brain iron dysregulation, morphological signs of early neurodegeneration and motor coordination deficits. *Neurobiol Dis* **81**, 119-133 (2015).
47. Lionel, A.C. *et al.* Disruption of the ASTN2/TRIM32 locus at 9q33.1 is a risk factor in males for autism spectrum disorders, ADHD and other neurodevelopmental phenotypes. *Hum Mol Genet* **23**, 2752-2768 (2014).
48. Verweij, K.J. *et al.* A genome-wide association study of Cloninger's temperament scales: implications for the evolutionary genetics of personality. *Biol Psychol* **85**, 306-317 (2010).
49. Drgon, T. *et al.* "Replicated" genome wide association for dependence on illegal substances: genomic regions identified by overlapping clusters of nominally positive SNPs. *Am J Med Genet B Neuropsychiatr Genet* **156**, 125-138 (2011).
50. Oh, Y. *et al.* Lin28B and miR-142-3p regulate neuronal differentiation by modulating Staufen1 expression. *Cell Death Differ* **25**, 432-443 (2018).
51. Kiebler, M.A. & DesGroseillers, L. Molecular insights into mRNA transport and local translation in the mammalian nervous system. *Neuron* **25**, 19-28 (2000).
52. Sutton, M.A. & Schuman, E.M. Dendritic protein synthesis, synaptic plasticity, and memory. *Cell* **127**, 49-58 (2006).
53. Martin, C.A. *et al.* Mutations in genes encoding condensin complex proteins cause microcephaly through decatenation failure at mitosis. *Genes Dev* **30**, 2158-2172 (2016).
54. Khan, T.N. *et al.* Mutations in NCAPG2 Cause a Severe Neurodevelopmental Syndrome that Expands the Phenotypic Spectrum of Condensinopathies. *Am J Hum Genet* **104**, 94-111 (2019).
55. Basu, S.N., Kollu, R. & Banerjee-Basu, S. AutDB: a gene reference resource for autism research. *Nucleic Acids Res* **37**, D832-836 (2009).
56. Consortium, C.-D.G.o.t.P.G. Identification of risk loci with shared effects on five major psychiatric disorders: a genome-wide analysis. *The Lancet* **381**, 1371-1379 (2013).

57. Samocha, K.E. *et al.* A framework for the interpretation of de novo mutation in human disease. *Nat Genet* **46**, 944-950 (2014).

58. Ji, X., Kember, R.L., Brown, C.D. & Bucan, M. Increased burden of deleterious variants in essential genes in autism spectrum disorder. *Proc Natl Acad Sci U S A* **113**, 15054-15059 (2016).

59. Ascano, M., Jr. *et al.* FMRP targets distinct mRNA sequence elements to regulate protein expression. *Nature* **492**, 382-386 (2012).

60. Darnell, J.C. *et al.* FMRP stalls ribosomal translocation on mRNAs linked to synaptic function and autism. *Cell* **146**, 247-261 (2011).

61. Watanabe, K., Taskesen, E., van Bochoven, A. & Posthuma, D. Functional mapping and annotation of genetic associations with FUMA. *Nat Commun* **8**, 1826 (2017).

62. Pocklington, A.J. *et al.* Novel Findings from CNVs Implicate Inhibitory and Excitatory Signaling Complexes in Schizophrenia. *Neuron* **86**, 1203-1214 (2015).

63. Abrahams, B.S. *et al.* SFARI Gene 2.0: a community-driven knowledgebase for the autism spectrum disorders (ASDs). *Molecular autism* **4**, 36 (2013).

64. Schizophrenia Psychiatric Genome-Wide Association Study, C. Genome-wide association study identifies five new schizophrenia loci. *Nat Genet* **43**, 969-976 (2011).

65. Pirooznia, M. *et al.* SynaptomeDB: an ontology-based knowledgebase for synaptic genes. *Bioinformatics* **28**, 897-899 (2012).

66. Bayes, A. *et al.* Characterization of the proteome, diseases and evolution of the human postsynaptic density. *Nat Neurosci* **14**, 19-21 (2011).
